# The multifaceted nature of Egyptian mummification: Paleoradiological insights into child mummies

**DOI:** 10.1371/journal.pone.0316018

**Published:** 2024-12-20

**Authors:** Stephanie Zesch, Stephanie Panzer, Alice Paladin, M. Linda Sutherland, Susanne Lindauer, Ronny Friedrich, Tanja Pommerening, Albert Zink, Wilfried Rosendahl

**Affiliations:** 1 German Mummy Project, Reiss-Engelhorn-Museen, Mannheim, Germany; 2 Department of Radiology, University Hospital Salzburg, Paracelsus Medical University Salzburg, Austria; 3 Institute of Biomechanics, Paracelsus Medical University, Salzburg, Austria; 4 Institute for Mummy Studies, Eurac Research, Bolzano, Italy; 5 MemorialCare Health Systems, Fountain Valley, California, United States of America; 6 Curt-Engelhorn-Centre Archaeometry gGmbH, Mannheim, Germany; 7 Institute of the History of Pharmacy and Medicine, Marburg University, Marburg, Germany; Hebrew University, ISRAEL

## Abstract

In accordance with ancient Egyptian beliefs, the preservation of the body after death was an important prerequisite for the continued existence of the deceased in the afterlife. This involved application of various physical interventions and magical rituals to the corpse. Computed tomography (CT), as the gold-standard technology in the field of paleoradiology, enables deeper insights into details of artificial body preservation. Therefore, CT was applied to investigate age at death, sex, mummification techniques, and the state of soft tissue preservation in 21 child mummies. The specimens are housed in European museums and were found in various archaeological sites. This sample included 12 males, 7 females, and two specimens of unknown sex, all between the ages of one and 14 years. Time periods were determined by radiocarbon dating, and time-related indicators of funerary equipment and decoration. Most of the mummies date from the Late Period to the Roman Period (664 BC–395 AD). Differences were identified concerning techniques of wrapping and embalming and removal of brain and internal organs, depending on age at death, social status and the archaeological site of the individuals. The variety and multifaceted nature of mummification and soft tissue preservation was analyzed in an integrated approach including a large number of specimens. The study highlights the significance of subadult remains as valuable bioarcheological archives to investigate burial customs and religious concepts in past societies.

## Introduction

The major foundation of ancient Egyptian beliefs and rituals concerning life after death was the desire for a continuing existence in the afterlife [1]. Preserving the corpse was essential, serving as the place of residence for the *ka* and the *ba*, both considered aspects of human existence, and to ensure the transformation of the earthly body into an eternal one [2]. Therefore, mummification was necessary to transform the corpse into a mummy and the deceased into a glorified spirit [3, 4]. The oldest document providing insights on the practice of mummification is the medical Papyrus Louvre-Carlsberg from the 18^th^ Dynasty (ci. 1450 BC). This papyrus includes a short passage on the removal of internal organs, followed by details on unguents and their application to the body, as well as bandages. These details were described in an unpublished PhD thesis that was made available to the authors by Sofie Schiødt **(S1 Text)**. The most comprehensive text is the *Embalming Ritual*, preserved on Roman Period papyri from the late 1^st^ to the early 2^nd^ century AD. The *Embalming Ritual* described how to treat and wrap the body parts and which spells were to be recited by the embalming priests [3, 4].

Based on the textual resources, it is known that the mummification procedure took about 70 days divided into two main periods. These periods were characterized by desiccating the body in the first half and applying aromatic embalming substances followed by wrapping the body in the second half [2, 4, 5]. As described in the literature [2, 5, 6], the most elaborate type of mummification included: 1) cleansing the corpse, 2) removing the brain (excerebration), 3) removing the viscera (evisceration), 4) dehydrating the corpse by the use of natron, 5) packing the body cavities with various materials, 6) anointing the body with aromatic substances, 7) remodeling missing body parts with artificial substitutes, 8) wrapping the corpse with bandages and sheets of linen, 9) and covering the corpse with amulets, jewelry, masks or other elements of adornment. Radiological studies, largely performed on mummies of adults, however revealed a great variety of artificial mummification techniques [7–11], depending on the time-period, local tradition, and social status of the deceased [6, 9].

Regarding subadults, various types of burials have been documented in excavations [12–15]. Fetuses, newborns, and infants up to circa 18 months were frequently buried within or close to settlements from around 3700 BC to the New Kingdom (1550–1070 BC) [13, 16]. For infants up to the age of 3 years, pottery vessels, amphoras and baskets were often used as funeral containers [13]. A well-known exception were two mummified fetuses placed in elaborately decorated coffins in the tomb of pharaoh Tutankhamun (1550–1295 BC) [16, 17]. Older children were usually buried in wooden boxes or coffins, interred in simple pits or tombs in cemeteries together with adults [13]. Research suggests that subadults were frequently mummified during the Ptolemaic and Roman Periods (332 BC–395 AD) [14, 16, 18]. This also supports the idea that in later time periods the mummification practice had become broadly accessible and less sophisticated than in the Pharaonic periods [14, 19, 20]. Previous radiological investigations primarily considered only a limited number of subadult mummies [20–23], thus, a detailed look into the mummified bodies of subadults in a large-scale study was a desideratum. Only two prior studies followed a comparative approach comprising a relatively large number of child mummies [11, 18]. The main result of a Master’s thesis was that the subadults frequently underwent similar preservation techniques as those observed in contemporary adults [18]. In a second study [11], no age or time-related patterns were revealed in terms of mummification techniques applied to the head. Fifty percent were eviscerated, and those showing preserved internal organs were aged 6 years or younger [11].

This study focuses on evidence of mummification methods and soft tissue preservation of 21 child mummies housed in seven museums in Switzerland, Germany, and Italy. Radiocarbon dating (^14^C analysis), computed tomography (CT) technology, and established methods of physical anthropology were applied. Results were evaluated in terms of time periods (primarily from the Late Period to the Roman Period, 664 BC–395 AD) [16], archaeological sites (especially Western Thebes and the Fayoum Oasis), and age at death of the specimens. A child dated from the Old Kingdom to the First Intermediate Period (2686–2055 BC) [16] was included for a comparative view. The current study demonstrates the variety of artificial mummification methods applied to the bodies of subadults in ancient Egypt, while considering several different variables. For the first time, it also provides insights into the state of soft tissue preservation of Egyptian child mummies though a systematic approach.

The following research questions were of particular interest:

- Which artificial techniques were applied to preserve the bodies?
- Were different methods of mummification used depending on the time period, geographical region, social status, and/or age at death?
- What state of preservation characterized the soft tissues?
- What effect did the applied techniques have on the preservation of the bodies?

## Materials and methods

### Materials

Twenty-one mostly intact bodies, whose state of preservation allowed for an accurate paleoradiological investigation, were included in this study. Information on the archaeological sites, burial contexts, antiquity traders, early archaeologists and collectors were published elsewhere [8, 24–29]. Following the recommendations of Squires and colleagues [30], the authors give an ethics statement referring to the guidelines underlying this research. The bodies were treated respectfully and studied carefully considering practical guidance and recommendations of German Museums Associations [31]. Sampling body tissues for radiocarbon dating was done in several mummies, however kept to a minimum. For a transparent presentation of our scientific data and to illustrate the results of our study, it was necessary to show a representative selection of CT reconstructions. We respect that there are individual and cultural differences in the ethical understanding and sensitivity of the visualization of human remains.

**Fig 1:**
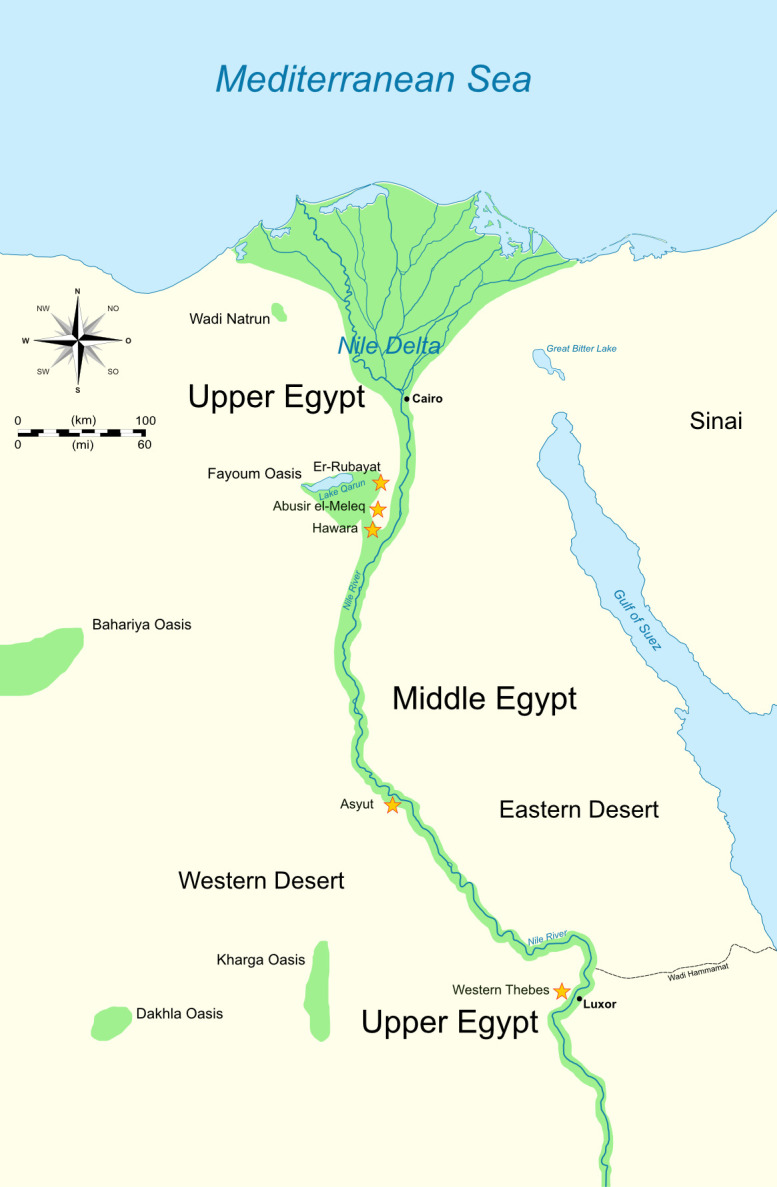
Map of ancient Egypt showing the sites from which the mummies have been obtained. The archeological sites are marked with yellow asterisks and Cairo was added as geographical reference city. Map (original): J Dahl, CC BY-SA 4.0, https://commons.wikimedia.org/wiki/File:Ancient_Egypt_map-en.svg, modified by S. Zesch.

Most of the individuals originated from Western Thebes (n = 8) and the Fayoum Oasis, including Hawara (n = 3), Abusir el-Meleq (n = 1), and Er-Rubayat (n = 1), (Fig 1, Table 1). Included are three mummies from a burial context known as the *Tomb of Aline*, dated from the 1^st^ to the 2^nd^ century AD in Hawara (Fayoum Oasis), and four mummies from the well-known Soter family, dated from the 2^nd^ century AD and buried in Theban Tomb (TT) 32 at the El-Khokha hill in Western Thebes. Both ensembles originated from elite Roman Period burials whose funerary equipment and decoration showed artistic characteristics from ancient Egyptian and Greek traditions [26, 32]. One mummy was presumed to be from Asyut (Middle Nile Valley), and for seven individuals, the archaeological sites were unknown. Five coffins were associated with six individuals. Two children from the Soter family (cases 1, 9) were buried in the same coffin. Six mummies were adorned with decoration such as a portrait (cases 5, 8, 19), a mask (cases 17, 20), and a net made of faience beads (case 21).

**Table 1:** The child mummies in the study.

Case	City/Country	Museum	Inv. No.	Name	Description	Sex	Age at death (years)	Age group (years)	Coffin/material	Archaeological site	Time period#
1	Berlin/DE	Ägyptisches Museum und Papyrussammlung	ÄM 505/04	Tkauthi/Ta-kau-udja	mummy with textile wrappings	F	1 (± 3 months)	neonate–1 year	present/wood	Western Thebes, El-Khokha, Soter family (TT 32)	Roman Period
2	Berlin/DE	Ägyptisches Museum und Papyrussammlung	ÄM 722	unknown	mummy with textile wrappings	M	1–1.5	1–3	absent	Western Thebes	Late Period–early Ptolemaic Period*
3	Berlin/DE	Ägyptisches Museum und Papyrussammlung	ÄM 704	Ta-scherit-en-pa-mai	mummy with textile wrappings	F	1.5–2.5	1–3	absent	Western Thebes	Late Ptolemaic Period*
4	Basel/CH	Naturhistorisches Museum Basel	III 8226	unknown	mummy with textile wrappings	Ind.	2–3	1–3	absent	unknown	Ptolemaic Period–Roman Period*
5	Berlin/DE	Ägyptisches Museum und Papyrussammlung	ÄM 11413	unknown	mummy with mummy portrait and textile wrappings	M	2–3	1–3	absent	Fayoum Oasis, Hawara, *Tomb of Aline*	Roman Period
6	Hildesheim/DE	Roemer- und Pelizaeus-Museum	PM 6356	unknown	mummy with textile wrappings	Ind.	2–4	1–3	absent	Asyut (?)	Old Kingdom–First Intermediate Period*
7	Berlin/DE	Ägyptisches Museum und Papyrussammlung	ÄM 505/03	Phaminis/Pa-Min	mummy with textile wrappings	M	2.5–4	1–3	present/wood	Western Thebes, El-Khokha, Soter family (TT 32)	Roman Period
8	Berlin/DE	Ägyptisches Museum und Papyrussammlung	ÄM 11412	unknown	mummy with mummy portrait and textile wrappings	F	2.5–4	1–3	absent	Fayoum Oasis, Hawara, *Tomb of Aline*	Roman Period*
9	Berlin/DE	Ägyptisches Museum und Papyrussammlung	ÄM 504/03	Sensaos/Ta-scherit-djed-Hor	mummy with textile wrappings	F	3–4	1–3	present/wood	Western Thebes, El-Khokha, Soter family (TT 32)	Roman Period
10	Leipzig/DE	Ägyptisches Museum—Georg Steindorff–der Universität Leipzig	6027	unknown	unwrapped mummy	M	3–4	1–3	absent	unknown	Roman Period*
11	Hildesheim/DE	Roemer- und Pelizaeus-Museum	PM 5206	unknown	mummy with textile wrappings	M	3–4	1–3	absent	unknown	Roman Period*
12	Frankfurt am Main/DE	Senckenberg Forschungsinstitut und Naturmuseum Frankfurt am Main	ÄS 18	unknown	mummy with textile wrappings	M	3–4	1–3	absent	unknown	Late Period–early Ptolemaic Period*
13	Turin/IT	Museo Egizio	Cat. 2230/1	Petamenophis/Padiamenemipet	mummy with textile wrappings	M	4–5	4–9	present/wood	Western Thebes, El-Khokha, Soter family (TT 32)	Roman Period
14	Berlin/DE	Ägyptisches Museum und Papyrussammlung	ÄM 16800/03	unknown	mummy with textile wrappings	F	4–6	4–9	present/cartonnage	Fayoum Oasis, Abusir el-Meleq	Roman Period
15	Berlin/DE	Ägyptisches Museum und Papyrussammlung	ÄM 723	unknown	unwrapped mummy	M	5–6	4–9	absent	Western Thebes	Third Intermediate Period–Late Period*
16	Frankfurt am Main /DE	Senckenberg Forschungsinstitut und Naturmuseum Frankfurt am Main	Ä 16 (IN 2463a)	unknown	unwrapped mummy	M	5–7	4–9	absent	unknown	assumed Ptolemaic Period–Roman Period
17	Berlin/DE	Ägyptisches Museum und Papyrussammlung	ÄM 12125	unknown	mummy with mummy mask and textile wrappings	F	6–7	4–9	absent	Fayoum Oasis, Hawara, *Tomb of Aline*	Roman Period*
18	Frankfurt am Main/DE	Senckenberg Forschungsinstitut und Naturmuseum Frankfurt am Main	Ä 17 (IN 2463b)	unknown	unwrapped mummy	M	7–9	4–9	absent	unknown	assumed Ptolemaic Period–Roman Period
19	Berlin/DE	Ägyptisches Museum und Papyrussammlung	ÄM 36101/01	unknown	mummy with mummy portrait and textile wrappings	F	9–10	4–9	absent	Fayoum Oasis, Er-Rubayat	Roman Period*
20	Frankfurt am Main/DE	Senckenberg Forschungsinstitut und Naturmuseum Frankfurt am Main	Ä 15 (IN 2462a)	unknown	mummy with mummy mask and textile wrappings	M	10–11	10–15	absent	unknown	assumed Ptolemaic Period–Roman Period
21	Basel/CH	Antikenmuseum Basel und Sammlung Ludwig	III 30	unknown	mummy with textile wrappings and a bead-net	M	12–14	10–15	present/wood	Western Thebes	Late Period–early Ptolemaic Period*

* = time period based on ^14^C data, # = final time period determined for the mummy

DE = Germany, CH = Switzerland, IT = Italy, Inv. No. = inventory number, TT = Theban Tomb, M = male, F = female, Ind. = indeterminate, cent. = century. Time spans are cited following Shaw [16]: Old Kingdom = 2686–2160 BC, First Intermediate Period = 2160–2055 BC, Third Intermediate Period = 1069–664 BC, Late Period = 664–332 BC, Ptolemaic Period = 332–30 BC, Roman Period = 30 BC–395 AD.

### Dating

Evidence on the time periods was obtained by using two approaches: 1) considering time-related indicators of funerary equipment (e.g., a related coffin or papyrus) and mummy decoration, and 2) radiocarbon dating (Table 1). Nine mummies were dated based on chronological indicators of the preserved funerary equipment or decoration. Radiocarbon dating was performed at the laboratory facilities of the Curt-Engelhorn-Centre Archaeometry in Mannheim (Germany). These analyses included 15 samples from 13 mummies: bone (n = 2), hair (n = 1), skin (n = 2), muscle (n = 1), and textiles (n = 9). The amount of bone required for radiocarbon dating is circa 1000 mg [33], whereas around 100 mg of sample is needed for the analysis of soft tissues, such as hair, skin, and textile [34]. Ancient Egyptian samples need to undergo an extra pretreatment step to remove potential contamination by foreign carbon, such as from conservation materials, resins, or bitumen-based embalming substances [35].

The following section summarizes the applied steps of pretreatment, decontamination, graphitization, and measurement of samples that are described in detail in several studies [36, 37]. Samples were first pretreated with the solvent benzene to remove possible bitumen. Thereafter they were processed by following an acid/base/acid (HCl/NaOH/HCl) sequence to remove contamination such as carbonates and humic acids. Bone samples underwent additional procedures to extract the bone collagen. To further eliminate contamination in the liquid collagen and to only date non-altered collagen, an ultrafiltration step was introduced before freeze-drying the sample [33]. After pretreatment and decontamination, samples were converted into CO_2_ gas by combustion in an elemental analyzer, and subsequently reduced into elemental carbon by specific graphitization machines. A small target with a graphite sample of circa 1 mg was prepared for measurement in an accelerator mass spectrometer (AMS). An AMS of type MICADAS [36] was used for the measurements of radiocarbon. These measurements comprise the samples of interest as well as measurements of standard samples, background samples and other quality control samples (e.g., IAEA standards with known ^14^C content). After measurement, the samples were calibrated into calendar dates by using a special software (OxCal) [38] with a regularly updated dataset IntCal (here IntCal20) [39]. Finally, the time period according to the ancient Egyptian chronology was determined by using the 2-sigma (95% probability) age-ranges calibrated ^14^C dates.

### Paleoradiological investigation

CT scanning was performed between 2009 and 2017 at different sites using scanners from different manufacturers (**S1 Table)**. There are no standardized imaging protocols published for the analyses of subadult archaeological remains [40]. The authors obtained permission to investigate the CT scans from the museums involved. Depending on the location of the museums, various medical centers provided stationary CT equipment in their hospitals to scan the mummies. Nine mummies were investigated by using a mobile CT scanner on a trailer, placed in front of the museum or at the museum depot. The following medical imaging software was applied to evaluate the CT scans: OsiriX (version 3.7.1, 64-bit, Pixmeo SARL, Geneva, Switzerland), RadiANT DICOM viewer (64-bit), and Horos (version 3.3.6, 64-bit, Horosproject.org). DeepUnity R20 XX (Dedalus HealthCare, Bonn, Germany) was used to apply a checklist and scoring system for the standardized assessment of the mummies’ soft tissue preservation [41].

### Age-at-death and sex estimation

Age at death was estimated by evaluating dental and skeletal markers with regards to the stage of tooth development and eruption [42–45], the presence and stage of fusion of cranial fontanelles (membranous space in the immature cranium), the development of primary ossification centers and the epiphyseal union of postcranial bones [46], as well as the maximum diaphyseal length of the long bones [47]. In two individuals (cases 7, 13), age at death mentioned on inscriptions of related coffins [48] was compared with the physical anthropological results. The specimens were sorted into age groups according to Wheeler [49] who considered 238 subadults from a Romano-Byzantine cemetery in the Dakhleh Oasis, Egypt. Categories were defined as: neonate–1 year; 1–3 years; 4–9 years, and 10–15 years. Those children whose possible maximum years of age overlapped with the minimum years of age of the next older age group were included in the younger age group (cases 1, 6–12, 19).

Sex determination was performed by identifying genitalia assessed by macroscopic observation in four unwrapped bodies, and radiologically (CT) in 17 mummies wrapped in bandages. The names of five individuals were inscribed either on a coffin, a related papyrus or on the wrappings. Evidence of sex could also be derived from iconographic details of a portrait, a mask or the decoration of a related coffin in ten mummies. In 11 mummies, the anthropological results were compared with evidence on sex derived from written names and sex-related characteristics of the mummy decoration.

### Evaluation of mummification and embalming techniques

Evidence of embalming was assessed by a visual check for dark-colored areas on the skin and/or textile wrappings, and radiologically through the presence/absence of radiopaque embalming substances on CT. Radiodensity of mummification materials and substances was based on the Hounsfield Unit (HU) scale. The possible identity of the mummification materials was suggested based on the evaluation of the obtained mean HU value on CT [50, 51]. CT scans were studied in terms of individual wrapping of the limbs, the presence of radiopaque embalming substances on/between the textile wrappings and on the skin, and for the composition of packing materials (granular materials, resin-like substances, textiles) present within the body cavities. CT scans were further evaluated regarding evidence of excerebration and the excerebration route (ethmoid, sphenoid, transorbital, cranio-cervical), and evidence of evisceration and the evisceration route (abdominal, perineal).

### Evaluation of soft tissue preservation

The applied checklist for the assessment of soft tissue preservation [41] included 97 defined checkpoints organized into two main categories: A. Soft tissues of the head and musculoskeletal system, and B. Organs and organ systems. A maximum score of 100 for each main category was possible, thus, a maximum total score of 200 was achievable for each specimen. The main categories A. and B. were further divided into subcategories of soft tissues. Main category A. comprises the subcategories A.1. Head and A.2. Musculoskeletal system, the latter being subdivided into A.2.1. Tendons and/or musculature, A.2.2. Peri- and intra-articular soft tissues, and A.2.3. Intervertebral discs. Main category B. includes the subcategories B.1. Central nervous system and peripheral nerves, B.2. Cardiorespiratory system, B.3. Gastrointestinal system, B.4. Genitourinary system, and B.5. Vasculature-arteries. Identified soft tissue checkpoints were scored and summed at the levels of the main categories A. and B.

Based on the soft tissue scores calculated for each individual, the summation scores were investigated in groups in terms of age at death (1–3 years, 4–9 years), evisceration (eviscerated/non-eviscerated), and radiological evidence of resin-like substances on the skin (present/absent). The aim was to reveal possible effects of the biological age and the applied mummification techniques on the mummies’ soft tissue preservation. The 1-year-old individual and the two specimens older than 10 years of age were not considered for this comparative analysis due to the low number of individuals of these ages included in the study.

## Results

### Dating

Most of the specimens (91%, 19/21) were dated from the Late Period to the Roman Period (664 BC–395 AD) [16]. The most ancient mummy (case 6) dated from the Old Kingdom to the First Intermediate Period (cal. BC 2397–2144, MAMS 24833) (Table 2). Radiocarbon dating of the two bone samples yielded a C/N ratio of 3.1 (MAMS 39364) and 3.6 (MAMS 31492), together with collagen yields of 12.4% (MAMS 39364) and 3.9% (MAMS 31492), indicating a good quality of bone collagen according to DeNiro [52]. Eight individuals were dated for the first time as part of this study (cases 2–4, 6, 10–12, 15). In three specimens, radiocarbon dating confirmed the timelines that had previously been presumed (cases 8, 17, 19). One individual (case 21) was wrapped in textiles dated from cal. BC 391 to 208 (MAMS 26278), however, the artisan decoration of the related coffin referred to the much earlier 21^st^ Dynasty (ci. 1069–945 BC) [16] indicating that the textiles wrapping, the body and the coffin were not from the same time period. In one specimen (case 2), the measured bone sample dated much later (cal. BC 396–228, MAMS 39364) than the textile sample (cal. BC 1617–1516, MAMS 39365) revealing reuse of the textiles. In the case of a female and her cartonnage coffin (case 14), samples showed older ages for both the wrappings (cal. BC 796–568, MAMS 37505) and the cartonnage material (cal. BC 354–114, MAMS 37504) than indicated by the female depicted on the coffin (Roman Period) (30 BC–395 AD) indicating reuse of both the textile and the cartonnage material.

**Table 2:** Radiocarbon dating and time periods.

Case	Inv. No.	*Radiocarbon dating (14C analysis)*							Time period+	Time period#
		*Lab*. *No*.	*Material*	^*14*^ *C age (BP) ± SD*	*Calibrated age (95% probability)*	*C/N ratio*	*Collagen yield (%)*	*C (%)*		
1	ÄM 505/04								2^nd^ cent. AD	Roman Period
2	ÄM 722	MAMS 39364	bone	2272 ± 18	cal. BC 396–228	3.1	12.4	44.7		Late Period–early Ptolemaic Period*
		MAMS 39365	textile	3302 ± 20	cal. BC 1617–1516			53.5		
3	ÄM 704	MAMS 37503	textile	2046 ± 19	cal. BC 105–cal. AD 22			43.3		Late Ptolemaic Period*
4	III 8226	Hd-28207	textile	2015 ± 18	cal. BC 49–cal. AD 60			n.a.		Ptolemaic Period–Roman Period*
5	ÄM 11413								1^st^–2^nd^ cent. AD	Roman Period
6	PM 6356	MAMS 24833	skin	3813 ± 26	cal. BC 2397–2144			37.2		Old Kingdom–First Intermediate Period*
7	ÄM 505/03								2^nd^ cent. AD	Roman Period
8	ÄM 11412	MAMS 30171	textile	1910 ± 22	cal. AD 65–210			39.4	1^st^–2^nd^ cent. AD	Roman Period*
9	ÄM 504/03								2^nd^ cent. AD	Roman Period
10	6027	MAMS 25890	skin	1911 ± 27	cal. AD 31–213			41.0		Roman Period*
11	PM 5206	MAMS 24831	hair	1975 ± 24	cal. AD 40–120			39.0		Roman Period*
12	ÄS 18	MAMS 22577	muscle	2247 ± 23	cal. BC 389–207			51.8		Late Period–early Ptolemaic Period*
13	Cat. 2230/1								2^nd^ cent. AD	Roman Period
14	ÄM 16800/03	MAMS 37504	textile (cartonnage coffin)	2163 ± 19	cal. BC 354–114			41.5	2^nd^ cent. AD	Roman Period
		MAMS 37505	textile (wrapping material)	2547 ± 20	cal. BC 796–568			40.3		
15	ÄM 723	MAMS 31492	bone	2429 ± 23	cal. BC 745–407	3.6	3.9	41.3		Third Intermediate Period–Late Period*
16	Ä 16 (IN 2463a)								assumed 3^rd^ cent. BC–1^st^ cent. AD [53]	assumed Ptolemaic Period–Roman Period
17	ÄM 12125	MAMS 30170	textile	1952 ± 22	cal. BC 24– cal. AD 128			40.3	1^st^–2^nd^ cent. AD	Roman Period*
18	Ä 17 (IN 2463b)								assumed 3^rd^ cent. BC–1^st^ cent. AD [53]	assumed Ptolemaic Period–Roman Period
19	ÄM 36101/01	MAMS 37509	textile	1941 ± 17	cal. AD 25–126			41.6	1^st^–2^nd^ cent. AD	Roman Period*
20	Ä 15 (IN 2462a)								assumed 2^nd^ cent. BC–1^st^ cent. AD [53]	assumed Ptolemaic Period–Roman Period
21	III 30	MAMS 26278	textile	2255 ± 20	cal. BC 391–208			39.7	21^st^ Dynasty	Late Period–early Ptolemaic Period*

= time period based on funerary equipment and decoration, * = time period based on ^14^C data, # = final time period determined for the mummy, Inv. No. = inventory number, Lab. No. = laboratory number, ^14^C age = conventional date, BP = before present, SD = standard deviation, BC = before Christ, AD = anno Domini, cal. = calibrated date, cent. = century, n.a. = not available, C/N = carbon-to-nitrogen ratio, C (%) = carbon yield. We presented C/N ratios and collagen yields (%) for the bone samples as well as C (%) for all samples. Time periods are cited following [16]: Old Kingdom = 2686–2160 BC, First Intermediate Period = 2160–2055 BC, Third Intermediate Period = 1069–664 BC, Late Period = 664–332 BC, Ptolemaic Period = 332–30 BC, Roman Period = 30 BC–395 AD.

### Age at death and sex

Age-at-death estimations revealed a range from neonate to 14 years of age (Table 1). Categorized into age groups, the sample included one neonate (maximum 1-year–old individual), 11 infants from 1–3 years, seven children from 4–9 years, and two juveniles from 10–15 years. In several subadults, the age estimations differed from those published earlier (cases 2, 7–9, [8]; 6 [25]; 10 [54]; 11 [55]; 20 [53]). Sex determination revealed 12 male and seven female individuals. Sex was not assessable in two mummies due to poor soft tissue preservation (cases 4, 6). In three specimens, sex determination differed from earlier investigations (cases 5, 15 [8]; 20 [53]). One mummy from the *Tomb of Aline* (case 5) was long time considered to be a male due to the position of the hands on the lower abdomen [26], however, later suggested to be female based on iconographic details of the related mummy portrait [56]. However, a penis and scrotum were identified on the latest CT investigation, confirming the mummy to be male.

### Textile wrappings and embalming of the bodies

**Fig 2:**
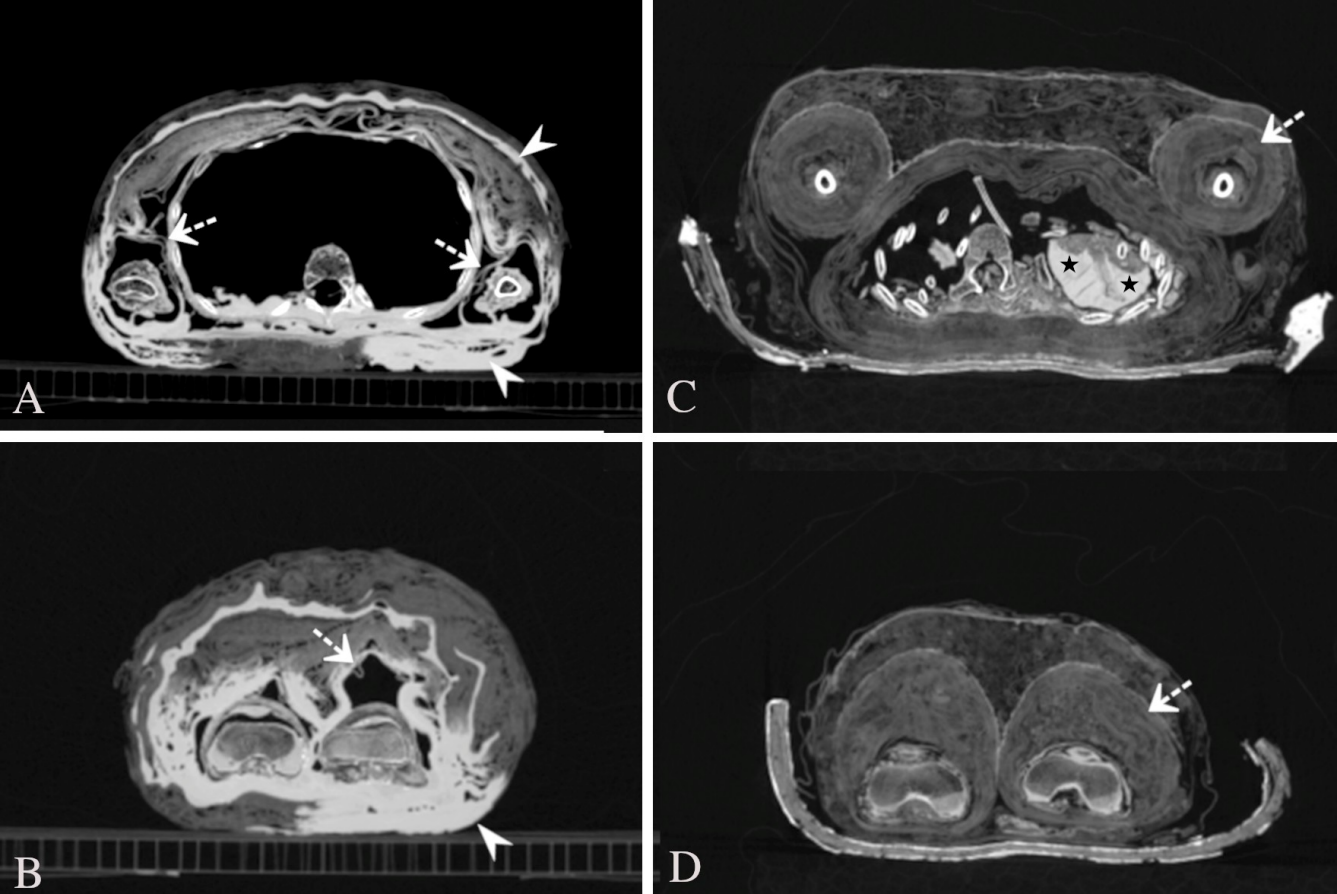
Individually wrapped limbs. (A and B) Mummy of a 3-to-4-year-old female individual called Sensaos (case 9), member of the Soter family, Western Thebes, Roman Period. Maximum intensity axial multi-planar reconstructions illustrate in (A), the arms (dotted arrows) and in (B), the thighs at the knee level (dotted arrow) individually wrapped by a few layers of textiles. Note the presence of resin-like substances (HU ≈ 153 ± 24.7), applied directly on the wrappings and between the layers of wrappings (arrowheads). (C and D) Mummy of a 4-to-6-year-old female individual inside a cartonnage coffin (case 14), Fayoum Oasis, Roman Period. Maximum intensity axial multi-planar reconstructions illustrate in (C), the torso and the arms and in (D), the thighs at the knee level individually wrapped by multiple densely packed layers of textiles (dotted arrows). Also note in (C) the presence of resin-like substances (HU ≈ 330 ± 64) inside the torso (asterisks).

Evidence on the techniques of artificial mummification utilized observed in this sample are listed in **S2 Table**. CT images demonstrating the mummification methods applied to each mummy investigated are included in **S1 File**. Textile wrappings were fully or partially preserved in 81% (17/21) of the mummies. It is assumed from the well-preserved soft tissues (some showed skin imprints from textiles and remnants of gilding) that the four unwrapped individuals were originally wrapped as well. In 41% (7/17) of the fully wrapped mummies, limbs had been wrapped individually by layers of textile before the body was fully covered by sheets and/or bandages (Fig 2A–2D). This type of limb wrapping was identified in almost all periods including the most ancient individual dated from the Old Kingdom to the First Intermediate Period (case 6). Limbs were individually wrapped in subadults from the elite Roman Period Soter family in Western Thebes, however not in the contemporary subadults from the upper-class family discovered in the *Tomb of Aline* in Hawara. Thickness and density of textile wrappings generally showed broad variation, ranging from a few loosely folded layers of textiles (case 9) to voluminously and densely packed layers (case 14).

**Fig 3:**
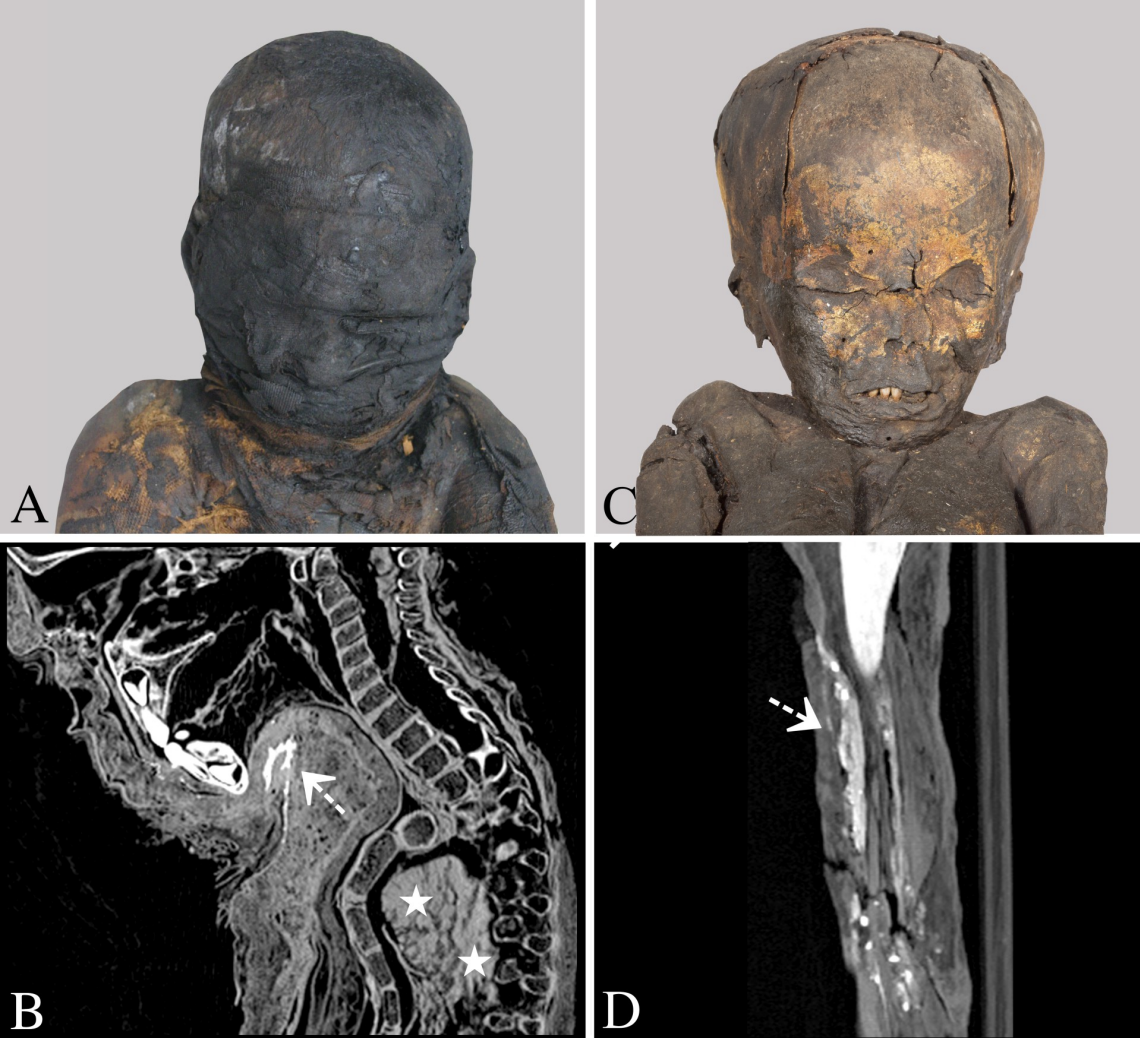
Evidence of embalming between the wrappings and on the skin. (A and B) Mummy of a 3-to-4-year-old male individual with textile wrappings (case 12), unknown archaeological site, Late Period to early Ptolemaic Period. The photo (A) shows evidence of dark-coloured embalming substances on the wrappings (© German Mummy Project, photo: S. Zesch). Maximum intensity sagittal multi-planar reconstruction (B) illustrates radiopaque substances with granular components between the textiles under the chin (dotted arrow). Also note the remnants of the dislocated liver inside the thoracic cavity (asterisks). (C and D) Unwrapped mummy of a 3-to-4-year-old male individual (case 10), unknown archaeological site, Roman Period. The photo (C) shows the dark-coloured skin probably caused by embalming substances and evidence of gilding on the face (© Ägyptisches Museum—Georg Steindorff—Universität Leipzig, photo: M. Wenzel). Thick-slab maximum intensity sagittal multi-planar reconstruction (D) illustrates areas of radiopaque substances with granular components on the right thigh (dotted arrows).

All 17 mummies with preserved wrappings showed evidence of various embalming substances applied on and/or between the textiles (Fig 3). These included dark-coloured parts in 71% (12/17) (Fig 3A), homogenous resin-like substances in 47% (8/17) (Fig 2A and 2B), and materials with granular components between the textiles in 53% (9/17) (Fig 3B). Twenty mummies showed macroscopic and radiological evidence indicating the use of embalming substances on the skin. These included granular materials in 70% (14/20) of the mummies (Fig 3D) and resin-like substances in 45% (9/20). Dark colouring on the skin was observed in 35% (7/20), however this analysis was limited to mummies whose wrappings were partially or fully absent (Fig 3C).

Nine mummies with radiological evidence of resin-like substances on the skin dated from the Third Intermediate Period (1069–664 BC) [16] or later. Most of them were discovered in Western Thebes (78%, 7/9). HU values measured for these homogeneously dense embalming substances between the wrappings, on the skin and inside the bodies of 13 mummies ranged between -216 and 401 (S2 Table). The minimum value of -216 for the embalming substances in one individual (case 19) was similar to beeswax with a value around 140 HU [51]. This individual was excluded from the calculation of mean HU value, as it deviates significantly from those HU values yielded for the 12 other mummies, with a range from 124 to 401. The mean HU value calculated for the homogeneous embalming substances is 207 ± 55 (n = 12) and thus similar to resin, published with a HU value about 71 ± 23.7 [50]. Granular materials between the textiles (Fig 3B), on the body surface (Fig 3D), and inside the body cavities of several mummies were not analyzed for their HU values, due to their different morphological structures and compositions, ranging from spot-like to uniformly dense materials.

### Excerebration, evisceration and packing materials

**Fig 4:**
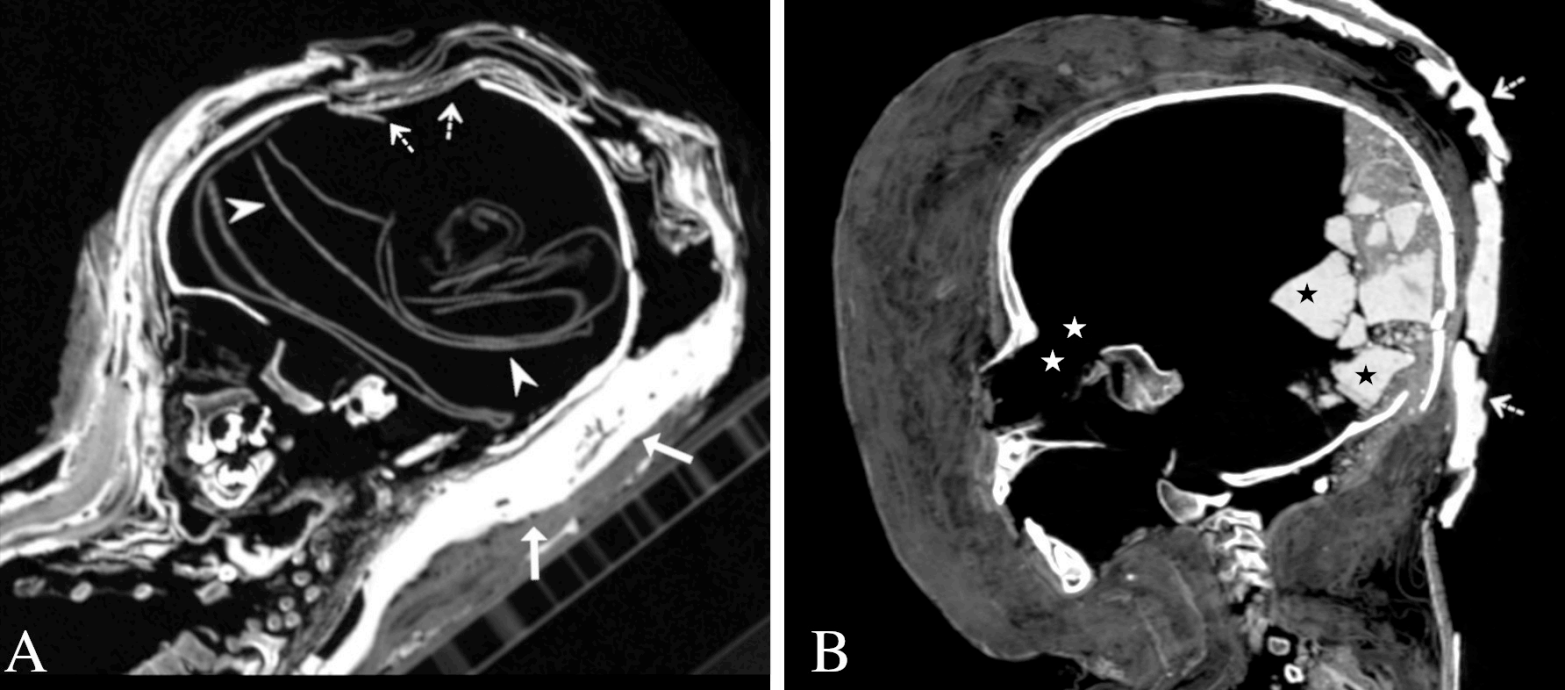
Excerebration and cranial packing. (A) Mummy of a 1-year-old female individual called Tkauthi (case 1), member of the Soter family, Western Thebes, Roman Period. Maximum intensity sagittal multi-planar reconstruction illustrates a defect in the left parietal bone (dotted arrows) and loosely folded textiles (HU ≈ -607.8 ± 91,5) inside the cranial cavity (arrowheads). Note the textile wrappings soaked with resin-like substances (HU ≈ 125 ± 87) behind the head (bold arrows). (B) Mummy of a 4-to-6-year-old female individual inside a cartonnage coffin (case 14), Fayoum Oasis, Roman Period. Maximum intensity sagittal multi-planar reconstruction visualizes a conglomerate of different radiopaque materials inside the cranial cavity, including resin-like substances (HU ≈ 401 ± 29) (black asterisks) probably inserted through the ethmoid route (white asterisks). Note the densely packed textile wrappings around the head anteriorly and portions of the cartonnage tray posteriorly (HU ≈ 830 ± 10) (dotted arrows).

Excerebration was observed in 67% (14/21) of the mummies and found in individuals from all age groups. The brain was not removed in the most ancient mummy from around 2200 BC (case 6), while both excerebrated and non-excerebrated children were identified in the later periods (664 BC–395 AD). All eight mummies from Western Thebes showed evidence of brain removal. Excerebration was not identified in 33% (7/21), including five specimens from the 1–3-year-age-group. Ninety-three percent (13/14) were excerebrated by perforating the ethmoid bone through the transnasal approach (Fig 4B). Three individuals with signs of transnasal brain removal also showed bone defects in the sphenoid, orbital or cranio-cervical regions. A 1-year-old female individual (case 1) revealed a rectangular defect in the ethmoid bone (maximum dimension of 2.9 cm) and a circular defect in the left parietal bone (maximum dimension of 5.2 cm) adjacent to the anterior fontanelle (Fig 4A). A few layers of loosely folded textiles were identified inside the skull. In 36% (5/14) of the excerebrated mummies, resin-like filling materials were found inside the cranial cavity, most probably entered through the nasal passage (Fig 4B). Nasal packings such as tampons made of textiles, were present in the nostrils of one male individual from Western Thebes (case 2).

**Fig 5:**
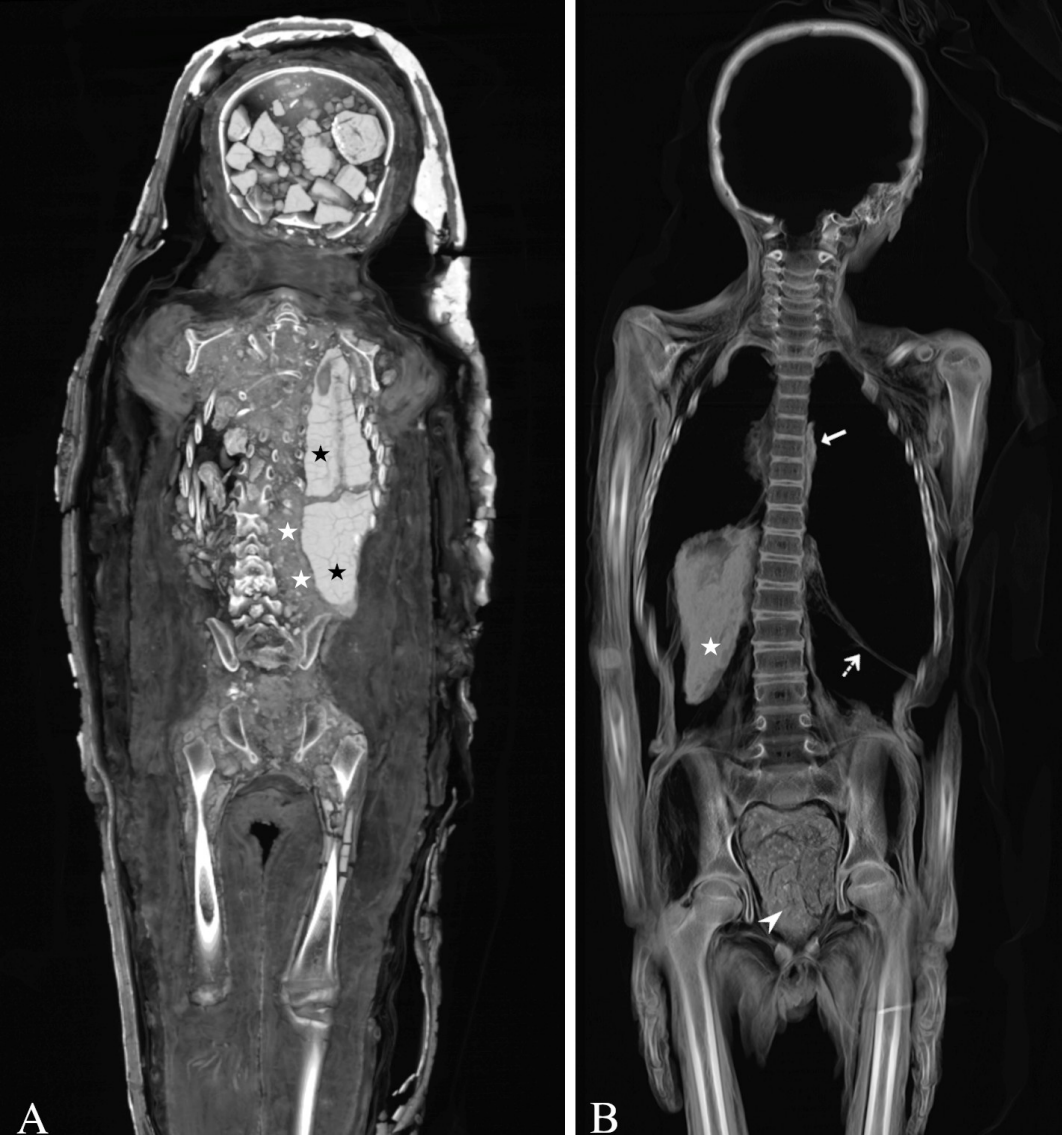
Torso packing. (A) Mummy of a 4-to-6-year-old female individual inside a cartonnage coffin (case 14), Fayoum Oasis, Roman Period. Thick-slab volume rendered coronal multi-planar reconstruction shows resin-like substances (HU ≈ 330 ± 64) (black asterisks) and granular materials (white asterisks) inside the torso. Resin-like substances are further visible inside the cranial cavity (HU ≈ 401 ± 29). Note that the lower limbs were individually wrapped with densely packed layers of textiles. (B) Unwrapped mummy of a 7-to-9-year-old male individual (case 18), unknown archaeological site, Ptolemaic Period to Roman Period. Thick-slab mean intensity coronal multi-planar reconstruction illustrates textiles inside the pelvis (arrowhead) inserted through the perineal route. Note the presence of the liver (white asterisk), the diaphragm (dotted arrow), and the preserved pericardium (bold arrow).

Evisceration was identified in 43% (9/21) of the mummies, including individuals from all age groups. It was not observed in 38% (8/21) of the subadults. In 19% (4/21) of the individuals, evisceration could not be determined due to the poor state of body preservation, which also holds true for the most ancient mummy (case 6). Both eviscerated and non-eviscerated subadults were identified from the Late Period onwards. As observed for the group of excerebrated mummies, a large proportion of eviscerated specimens (78%, 7/9) were from Western Thebes. Two non-eviscerated individuals with a very elaborate decoration style were from Hawara (cases 8, 17). Regarding the two individuals from the 10–15 years age group, excerebration and evisceration was evidenced in one individual from Western-Thebes (case 21), however not in the other specimen from an unknown archaeological site (case 20). Internal organs were more frequently removed in children from 1–3 years (n = 6) than in subadults from 4–9 years (n = 3). In 56% (5/9) of the mummies, this was achieved through an incision in the left side of the lower abdomen. In one individual (case 2), the perineal route was most likely used for evisceration and to insert textile rolls, loose textiles and resin-like embalming substances in the abdomen/pelvis. The presence of packing materials inside the torsos of three eviscerated specimens (cases 3, 14, 21) indicated the removal of internal organs, however, the evisceration route could not be determined due to the poor state of preservation. Mummification materials, such as resin-like embalming substances, textiles, or granular materials were found in all eviscerated mummies and usually identified as a mixture of various materials (Fig 5A). In two mummies (cases 17, 18), several organs, e.g., lungs, heart, and liver were preserved inside the bodies, although textiles and granular materials were inserted into the pelvis through the perineal route (Fig 5B).

### Soft tissue preservation

The detailed results obtained from the application of the soft tissue checklist and scoring system are given in **S3 Table**. Shrunken remnants of the brain were identified in 57% (12/21) of the mummies. Brain remnants were also found in five individuals with evidence of brain removal, indicating that brain tissue was not always fully removed. The eight non-eviscerated mummies showed that additional parenchymal tissues were frequently preserved, such as the brain (7/8 non-eviscerated mummies), the lungs (right 7/8, left 8/8), the pericardium (7/8), the diaphragm (right 6/8, left 7/8), the tongue (6/8), the liver (8/8), and the kidneys (right 5/8, left 5/8). However, hollow organs with thin membranes were rarely identified, such as the esophagus (0/8), the stomach (0/8), the gallbladder (0/8), and the urinary bladder (1/8). Shrunken remnants of the spleen were preserved in three mummies, and remnants of the pancreas were not observed at all. Remnants of vasculature and arteries were identified in only five mummies. Concerning the nine eviscerated mummies, organs of the cardiorespiratory system (lungs, pericardium, diaphragm), organs of the gastrointestinal system (stomach, liver, pancreas, intestines) and organs of the genitourinary system (kidneys, urinary bladder, prostate) had been mostly removed.

**Fig 6:**
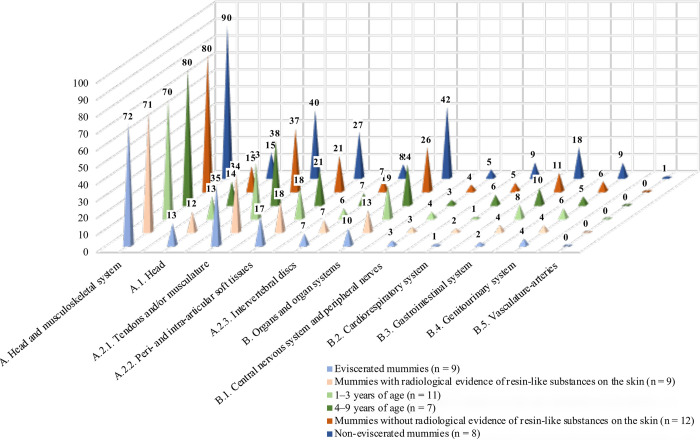
Results of the mean soft tissue scores calculated in groups in terms of age at death, evisceration and radiological evidence of resin-like embalming substances on the skin. The figure represents mean scores calculated for the main categories A and B, and for the subcategories. Subcategory A.2. (musculoskeletal system) is not visualized as a single row in the figure, as this subcategory is further subdivided (and represented) by the categories A.2.1., A.2.2., and A.2.3. Note that 7/9 eviscerated mummies showed radiological evidence of resin-like substances on the skin, while 7/8 non-eviscerated mummies lacked radiological evidence of resin-like substances on the skin.

The total soft tissue preservation scores (**S3 Table)** ranged from 24.0 (soft tissues were poorly preserved) (case 6) to 145.5 (soft tissues were frequently identified) (case 12), with a mean total score of 96. The evaluation of the two main categories resulted in a mean score of 76 for category (A), Soft tissues of the head and musculoskeletal system, and a mean score of 20 for category (B), Organs and organ systems. However, the mean score obtained for the organ and organ systems was influenced by the absence of internal organs in eviscerated mummies (Fig 6). Indeed, soft tissues of the head and the musculoskeletal system showed generally higher mean scores than for organ tissues. The group of 4–9-year-old individuals had slightly higher mean scores than mummies of infants. Interestingly, soft tissues of the head and the musculoskeletal system showed higher scores in mummies without resin-like substances on the skin compared with mummies with resin-like substances on the skin. Non-eviscerated mummies, which almost entirely lacked radiological evidence of resin-like substances, with one exception (case 15), revealed the highest soft tissue scores in this sample.

## Discussion

### Dating

Older textiles were reused for wrapping of the bodies in two mummies (cases 2, 14). In one specimen (case 14), the cartonnage material of the coffin was also made from older textiles. The reuse of bandages and cloths for wrappings and the reuse of older materials for cartonnage coverings (usually made of compressed and stuccoed textiles or papyri) have been previously reported [2, 19]. One male individual from Western Thebes, dated from the Late Period to the early Ptolemaic Period (case 21), was placed inside a decorated 21^st^ Dynasty (1069–945 BC) [16] coffin. As the mummy and the coffin were acquired in Egypt, and donated to the museum in 1840, it is unclear whether this ensemble was originally found together in the same tomb. This individual could have been placed inside the 21^st^ Dynasty coffin either by the embalmers or may have been placed in the coffin in the early 19^th^ century to increase its appeal for the antiquities market. Tombs and coffins, the latter most evident in the Third Intermediate Period (1069–664 BC) [16] were often reused for burials [57], as described by different scholars [58–60]. Thus, a recombination of a mummy/coffin ensemble in ancient Egyptian times should be considered as well.

### Age at death and sex

This sample was not appropriate for a demographic evaluation, as the individuals are not from the same geographical region and time period. For Phaminis (case 7), the age-at-death estimation, based on the dental status, developmental stage of epiphyses, and long bone length, suggested 2.5–4 years, and thus differs from the 7–8 years determined in a previous study [8]. Different translations for the age at death on the coffin were found in the literature [8, 48, 61]. The latest translation of the lifetime of Phaminis suggested “1 year, 10 months, 18 days” for the Demotic and “2 years…” for the Greek inscription [62]. Due to the previously assumed higher age at death compared to the inscription on the coffin, Germer and colleagues [8] concluded that this was not the mummy of Phaminis. However, the age at death suggested in this investigation (2.5 to 4 years) is similar to the age written on the coffin (2 years). The estimated age at death, the densely packed layers of textiles and the huge amount of resin-like embalming substances, both known from other Soter family members [3, 7, 24, 63–67], indicated that this is most likely the mummy of Phaminis. Additional discrepancies were found in terms of body measurements, age at death and sex during the recent analyses of the male Phaminis (case 7) and the female Sensaos (case 9), both Soter family members. Thus, these specimens were reidentified in that the mummy of Phaminis was renumbered with ÄM 505/03 (previously 504/03) and the mummy of Sensaos with ÄM 504/03 (previously ÄM 505/03).

### Textile wrappings and embalming of the bodies

Mummies from almost all time periods showed the practice of wrapping limbs individually before the body was fully covered by textiles. This technique, applied to preserve the anthropomorphic shape of the deceased, was practised from the Old Kingdom (2686−2125 BC) [16] onwards, handed down in written sources and verified in other mummy examinations [3, 4]. While limbs had been individually wrapped in all subadults from the Roman Period upper-class Soter family in Western Thebes, this practice was not identified in the contemporary upper-class children from the *Tomb of Aline* in Hawara, indicating that different techniques were applied according to local tradition, even in individuals from the same time period and social status. The coexistence of different mummification methods was also described by the Greek historian Herodotus of Halicarnassus (ci. 484–425 BC) [6]. In this current study, mummies were apparently more frequently treated with resin-like substances in Western Thebes than in the Fayoum Oasis, indicating local differences in the use of embalming substances.

Due to the lack of biomolecular analyses, this study did not permit conclusions on the precise composition of the resin-like substances found during the CT examinations. Embalming agents in the mummification procedure had both religious functions associated with the “odour of the god” [2] and preservative functions [2, 5, 6, 68]. They were applied for their antifungal, antibacterial, and waterproofing properties [6, 69]. From the 22^nd^ Dynasty (945–715 BC) [16] onwards, bitumen was increasingly used for its liquid-repellant properties [6], the assumed effect to make bodies more rigid and to fix textile layers [19]. Biomolecular methods revealed the presence of fragrant or antiseptic oils, tars, and resins in vessels discovered in an embalming workshop in Saqqara dated from the 26^th^ Dynasty (664–525 B.C.) [70]. A Graeco-Roman Period mummy head showed residues of resin, most likely natural resin, pitch, or tar, from the Pinaceae family conifer, together with fat, oil, or wax [71]. These findings correspond with the *Embalming Ritual*, preserved on Theban papyri from the 1^st^ to the 2^nd^ century AD, which described the use of natron, bitumen, and aromatic plants to embalm the bodies during this particular time period [4, 6].

### Excerebration, evisceration and packing materials

The result of 67% excerebrated subadults in this study is similar to a prior study which showed excerebration in 50% of the children [11]. Almost all of the excerebrated mummies had a perforation in the ethmoid bone, and occasionally in the adjacent cranial bones. The perforation was most likely made by a metal instrument with a curved end, such as usually applied for the extraction of the crushed liquefied brain [72]. Filling materials inside the cranial cavity (resin-like substances, textiles) and inside the nostrils (tampons made of textiles) were rarely identified. These findings are similar to those described in Loynes [11] where cranial packing was observed in 8% of the subadults and therefore much less frequently than in adults. It is plausible to assume for the 1-year-old specimen (case 1), that the brain was removed through the ethmoid defect. The thin layers of textiles inside the cranial cavity were probably introduced through the parietal defect, as the ethmoid defect was too small to insert and unfold textiles. The authors assume that the less frequent brain removal in children below the age of 3 years was to prevent the fragile facial anatomy of infants from collateral damage during the attempt at brain extraction. As all children from Western Thebes showed signs of excerebration, this procedure was likely performed more frequently in the workshops of Theban embalmers.

Eviscerated and non-eviscerated mummies in this study were identified in approximately equal numbers, which was similar to an earlier study [11] with a proportion of 50%. As 75% of the adults considered in that study [11] were eviscerated, this indicated that internal organs were more frequently removed in adults than in subadults. As suggested by Davey and colleagues [20], the use of desiccating and embalming agents may have accelerated the mummification of children compared to adults. In our study, an incision in the left abdominal wall was most frequently used for evisceration, however, the perineal route was used occasionally. The presence of resin-like substances, textiles, and granular materials correlated with findings described in the literature, such as aromatic resins, linen, sawdust, sand, mud, chaff, chopped straw, lichen, onions [5]. In the case of granular materials observed inside the body cavities, natron left from the dehydration treatment should also be considered. Various mummification methods were also observed in human remains dated from the late New Kingdom to the Late Period, discovered in Dra’ Abu el-Naga, Western Thebes [60]. Morphological analyses of mummies from this earlier time-period revealed excerebrated and eviscerated mummies, packing materials (e.g., sawdust, plants), embalming substances (presumably resin, mud, oil, wax), partly resin-soaked linen wrappings, as well as non-excerebrated and non-eviscerated specimens [60].

Our initial analysis suggested that evisceration was performed more frequently in children younger than 3 years, contrasting with Loynes [11] where evisceration was not observed in children younger than 6 years. However, a more frequent removal of internal organs in children younger than 3 years did not seem plausible considering that the brain was less frequently removed in the same age group in our study. In addition to age at death, provenience was utilized as another relevant factor to study the evisceration in children younger than three years. Interestingly, most of the eviscerated and all excerebrated mummies were from Western Thebes, suggesting that local practises played a role in the use of a specific mummification technique as described elsewhere [9, 10, 65]. The absence of evisceration in two elaborately wrapped and decorated mummies from the *Tomb of Aline* does not necessarily indicate a low-quality mummification, as evidenced from other studies as well [73, 74].

### Soft tissue preservation

Previously the checklist and scoring system had been applied on only four Egyptian mummies, two of which were rather poorly preserved [74, 75]. Due to this lack of comparative data from ancient Egypt, the authors referred to a study on anthropogenic and naturally preserved mummies from the Capuchin crypt in Palermo, Italy, dating from the late 18^th^ to the late 19^th^ century AD [76]. The rare identification of hollow organs in the eight non-eviscerated subadults in this study was also described for the Palermo mummies, where hollow organs were also often collapsed and could only be identified as thin membranes [76]. Our examination further revealed that shrunken parenchymal organs were altered significantly in size, shape, and position, however were mostly well-preserved, similar to the Sicilian crypt mummies. This was not observed for the spleen (rarely identified) and the pancreas (absent), which both require embalming shortly after death to prevent the autolysis process [68, 76]. The poor detectability of vasculature/arteries was probably caused by both the shrinkage of soft tissues and the limited ability to visualize these delicate anatomical structures using conventional CT technology, especially in subadults.

The better preservation of the soft tissues of the head and musculoskeletal system compared to the organs and organ tissues has also been observed in other studies [68, 76, 77]. As suggested by Lynnerup [77], this was probably caused by the larger ratio of skin surface area relative to the underlying body volume. This allowed better evaporation of water through the skin of the head, extremities, fingers, and toes [77]. As dehydration causes only minor changes in the shape and structure of tendons, ligaments, cartilage, dura mater and intervertebral discs, the proximity of these connective tissues to the skeleton often facilitates their identification [76].

The slightly higher mean scores of soft tissues observed for the group of 4–9-year-old individuals indicated a slightly better state of preservation compared to the 1–3-year-old infants. This possibly resulted from their larger stature and physical robustness against external influences. The higher values calculated for the soft tissues of the head and the musculoskeletal system in mummies without resin-like substances on the skin suggested that these tissues were better preserved, or easier to identify than in mummies extensively treated with resin. It remained unclear whether this was caused by the extensive use of resin-like substances or by radiological limitations in the soft tissue identification due to resin leakage. The high soft tissues scores observed for non-eviscerated mummies, which mostly did not undergo a resin-like body treatment, indicated that this group of mummies were the best-preserved in the sample. These observations confirmed the significance of natron to absorb water from the soft tissues and the use of textile wrappings to protect the body externally. It was suggested by Panzer and colleagues [78], that the bodies of two poorly preserved subadults (cases 14, 19) from the nearby Fayoum Oasis were probably laid inside a bath of liquid natron instead of applying solid natron to achieve soft tissue dehydration.

## Conclusions

This interdisciplinary comparative study obtained detailed results into mummification techniques and soft tissue preservation in Egyptian child mummies, mostly from the Late Period to the Roman Period. Radiocarbon dating applied on various types of materials accompanied with the body can help to shed light into the diversity of burial customs. As merely dating textiles might not necessarily date the mummies themselves, dating body tissue is therefore generally preferable. New results concerning age at death and sex of the individuals underscore the benefit of re-examining specimens using state-of-the-art imaging techniques.

This study demonstrates the variety of mummification methods applied to the bodies of subadults. It further reflects the scope of factors, such as age at death, social status/wealth of the family, and local differences, that played a role in the choice of a particular technique. Different mummification methods were applied in the same time period. The brain was less frequently removed in children younger than three years. Brain and internal organs were removed more regularly and resin-like embalming substances were more frequently applied in Western Thebes than in the Fayoum Oasis. The checklist and scoring system applied for a systematic assessment of soft tissues revealed insights into the state of soft tissue preservation with regard to age at death, evidence of evisceration and the use of resin-like embalming substances. Soft tissues of the head and the musculoskeletal system were better-preserved, or easier to identify, in children older than three years and in bodies without radiological evidence of resin-like embalming substances. The use of large amounts of resin-like embalming substances, particularly applied in Western Thebes, made the paleoradiological identification of soft tissues more difficult and probably worsened the state of preservation.

Methodological limitations arose in the identification of delicate soft tissues, particularly vasculature/arteries, which may be resolved by the selective use of high-resolution imaging techniques (e.g., micro-CT). The composition of embalming substances and identity of other mummification materials could be analyzed in more detail using biochemical and microscopic methods. This would require sampling of body tissues and materials which was often not possible and also not intended to be part of this radiologically focused study. Similar systematic studies considering a broader range of mummies from different periods and sites will further deepen our understanding of the burial customs applied to subadults in ancient Egypt.

## Supporting information

S1 Text List of used unpublished works. (DOCX)

S1 Table Summary of CT scan parameters. kV = kilovolt, mA = milliampere, mm = millimeter. Two CT scans with different parameters were generated in cases 4, 10 and 13.
(XLSX)

S2 Table Evidence on the applied mummification techniques. Inv. No. = inventory number, TT = Theban Tomb, M = male, F = female, Indet. = indeterminate, D = dark-coloured areas, Res = resin-like substances, Gr = granular materials, T = textile, Int = intact, Dis = disrupted, E = ethmoid, Sp = sphenoid, TO = transorbital, CC = craniocervical, Abd (left) = abdominal incision (left flank), per = perineal. Time spans are cited according to [16]: Old Kingdom = 2686–2160 BC, First Intermediate Period = 2160–2055 BC, Third Intermediate Period = 1069–664 BC, Late Period = 664–332 BC, Ptolemaic Period = 332–30 BC, Roman Period = 30 BC–395 AD. Abbreviations used to shorten the mummification techniques are taken from [11].
(XLSX)

S3 Table Results of the radiological checklist and scoring system applied for the assessment of soft tissue preservation. The checkpoints are evaluated with “+” (structure is present) or “–”(structure is absent). Values of detected checkpoints were summed at the levels of the subcategories, the main categories and the total score. Values of checkpoints that are not present are given in brackets.
(XLSX)

S1 File Catalogue with images from each mummy investigated. (PDF)
