# Supplementary material for: The multifaceted nature of Egyptian mummification: Paleoradiological insights into child mummies
Source: PLoS One. 2024 Dec 20;19(12):e0316018. doi: 10.1371/journal.pone.0316018 (PMC11661624; doi:10.1371/journal.pone.0316018)
Supplement: S1 Text — (DOCX) [file pone.0316018.s001.docx]

**S1 Text. List of used unpublished works**

Schiødt S. Medical science in ancient Egypt: a translation and interpretation of Papyrus Louvre-Carlsberg (pLouvre E 32847 + pCarlsberg 917). University of Copenhagen. PhD thesis, 2020.

By the courtesy of Sofie Schiødt
