# Supplementary material for: The multifaceted nature of Egyptian mummification: Paleoradiological insights into child mummies
Source: PLoS One. 2024 Dec 20;19(12):e0316018. doi: 10.1371/journal.pone.0316018 (PMC11661624; doi:10.1371/journal.pone.0316018)

S1 File. Catalogue with images from each mummy investigated.[1]

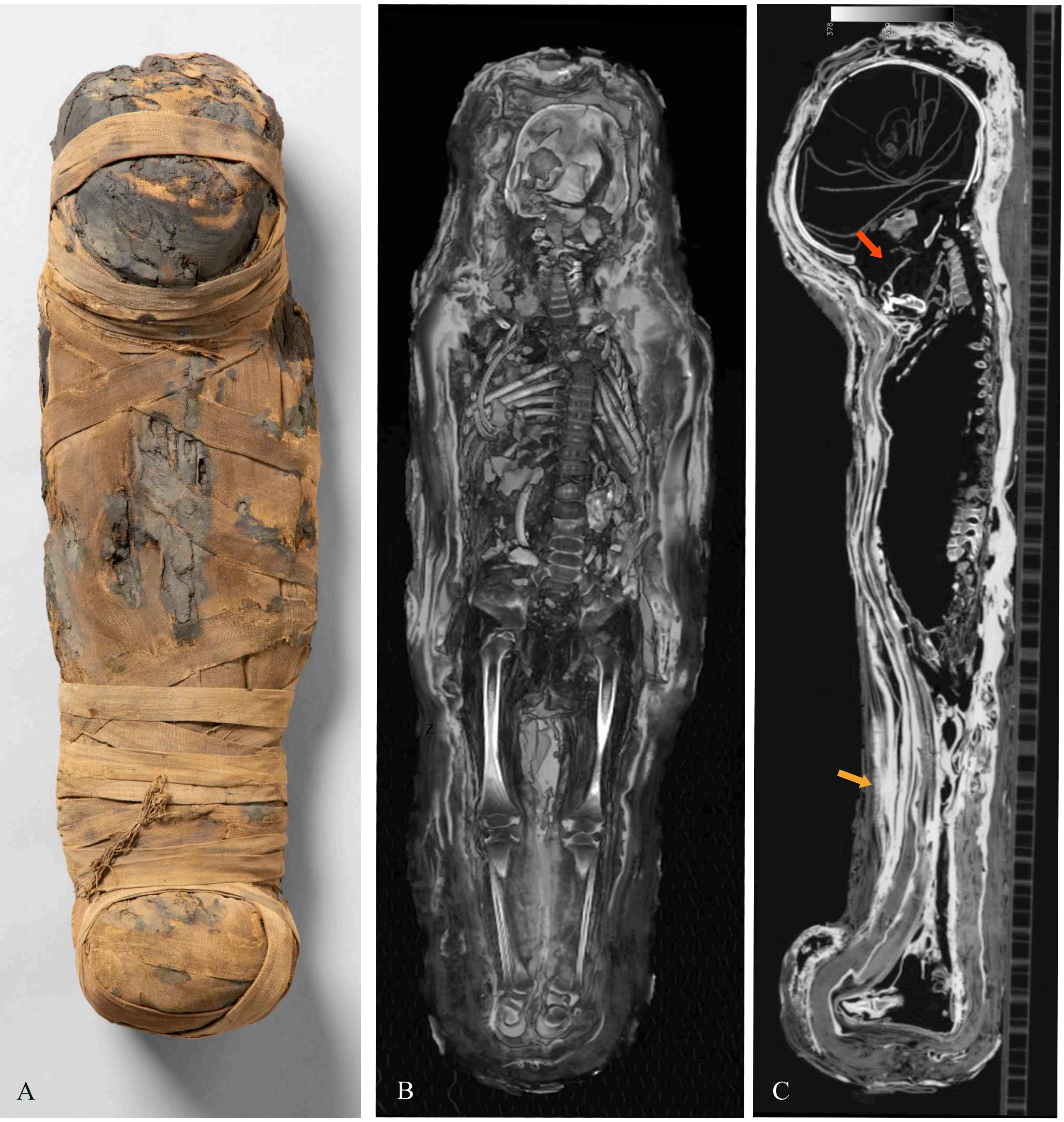

Case 1 (ÄM 505/04) - Western Thebes, Roman Period, 1-year-old female individual.

(A) Dark-colored areas of textiles indicate the use of embalming substances between the various textile layers (© SMB - Ägyptisches Museum und Papyrussammlung, photo: S. Steiß). **(B) Coronal plane** - Several bones of the skull and torso are disarticulated and fragmented. **(C) Sagittal plane** - The brain was removed through the ethmoid (red arrow). Few layers of loosely folded textiles are visible inside the cranial cavity. Textile wrappings are soaked with resin-like embalming substances (orange arrows). **(D) Axial plane** - Note the eviscerated torso and embalming substances (orange arrows) between the layers of textiles (CT image reconstructions: © German Mummy Project, S. Zesch).

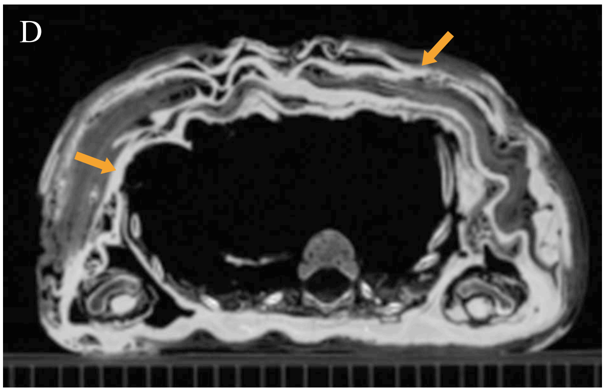

[1]Photos representing the mummies from the Ägyptisches Museum und Papyrussammlung in Berlin are reproduced by the courtesy of Prof. Dr. Friederike Seyfried (museum director).

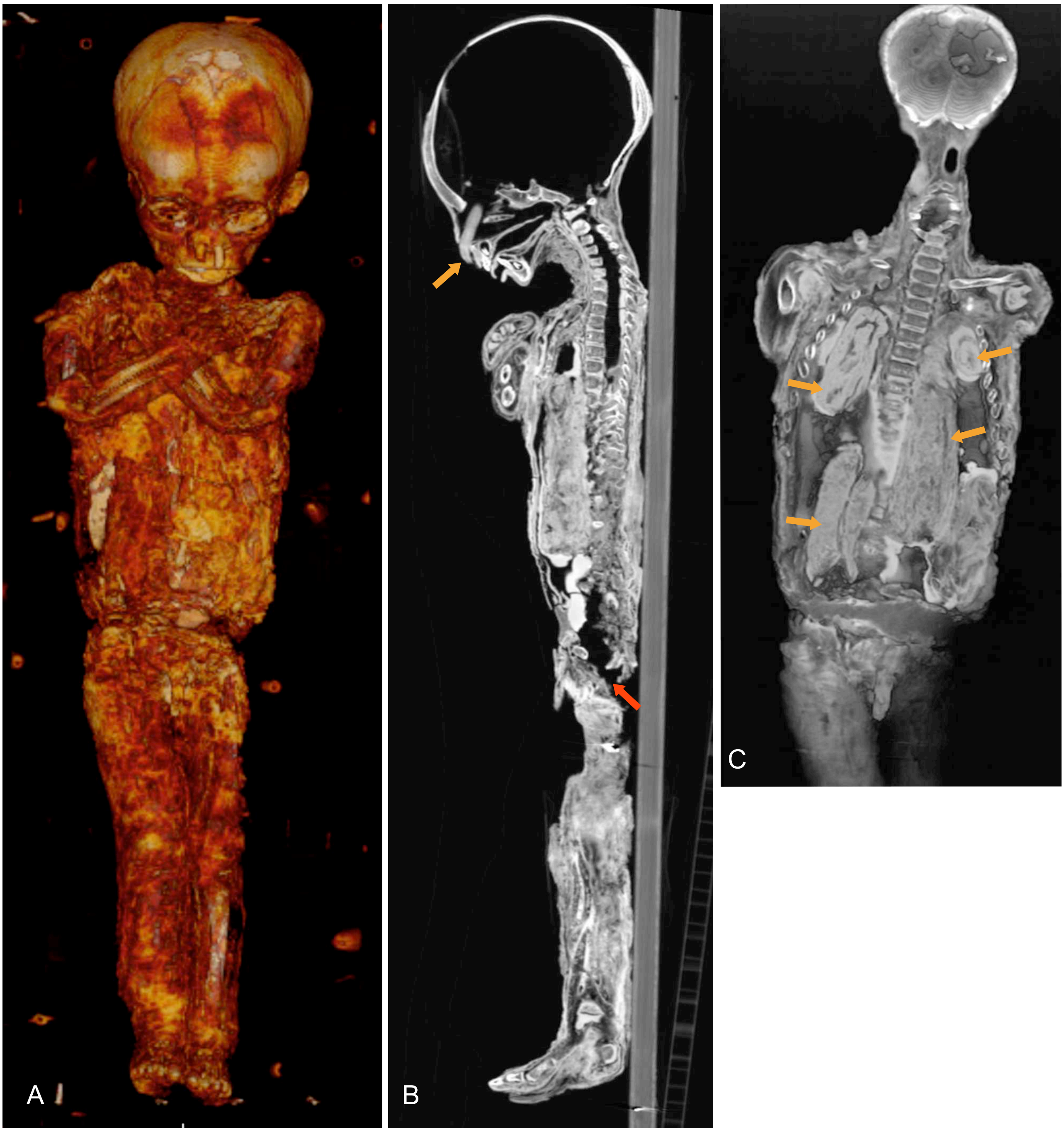

Case 2 (ÄM 722) - Western Thebes, Late Period to early Ptolemaic Period, 1-to-1.5-year-old male individual.

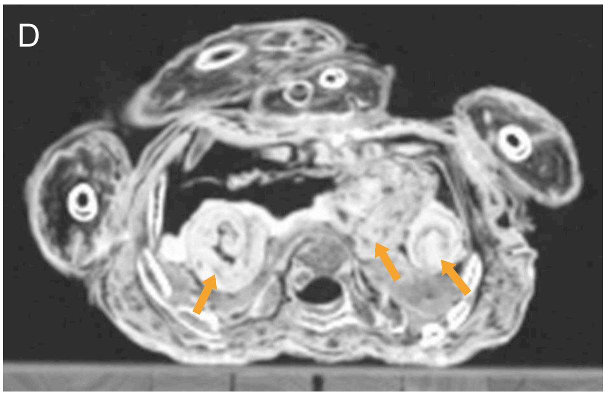

(A) 3D-Volume rendered reconstruction illustrates the mostly unwrapped mummy with the torso damaged at the level of the abdomen/pelvis. **(B) Sagittal plane** - The brain was removed through the ethmoid. Tampons made from textiles were inserted into the nostrils (orange arrow). The perineal route (red arrow) was most likely used for evisceration and to insert textile rolls and resin-like embalming substances. **(C) Coronal plane** - Longitudinal rolls of textiles (orange arrows) are present inside the torso surrounded by resin-like embalming substances. **(D) Axial plane** - Note three of the textile rolls (orange arrows) inside the thoracic cavity (CT image reconstructions: © German Mummy Project, S. Zesch).

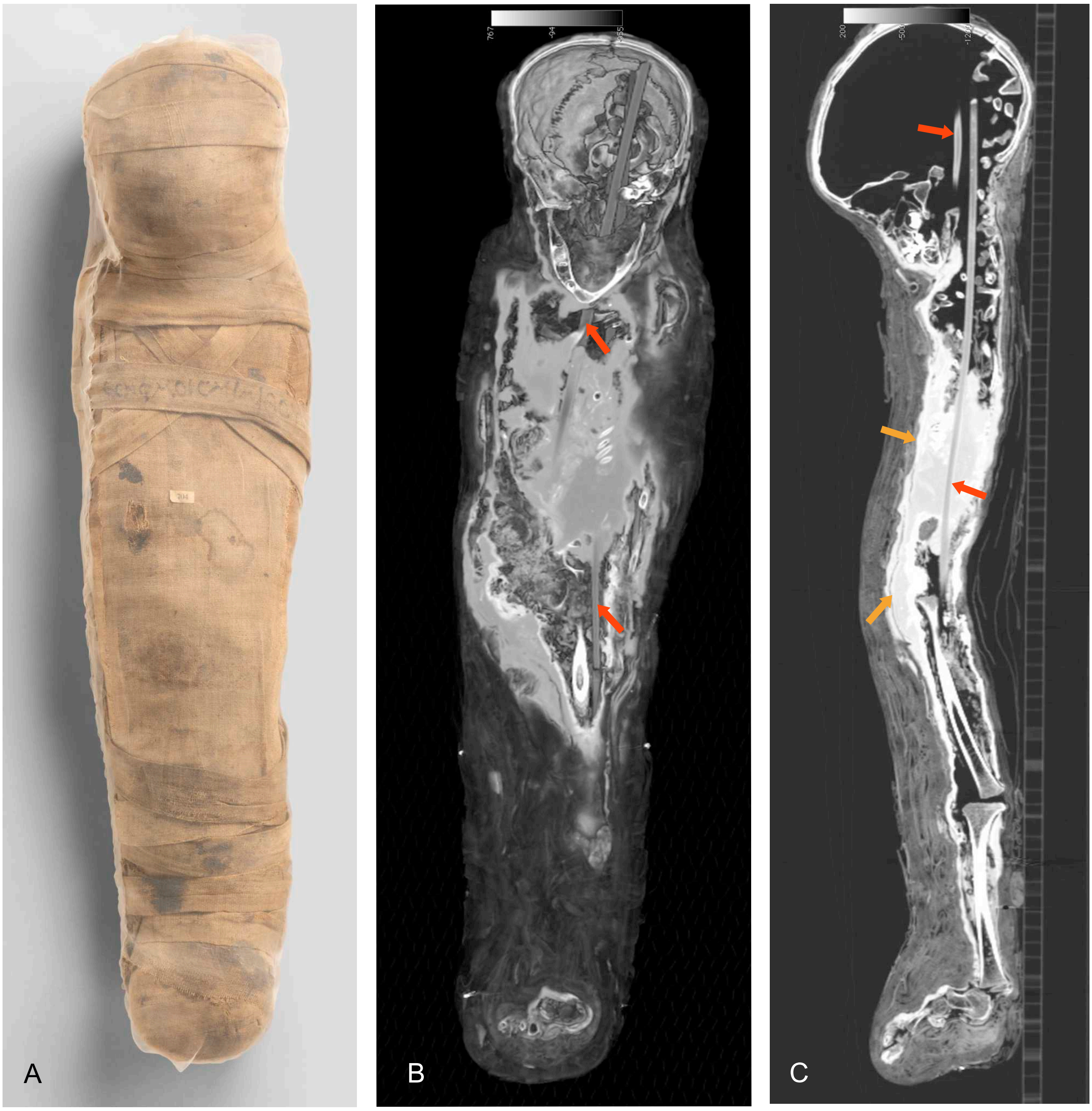

Case 3 (ÄM 704) - Western Thebes, Late Ptolemaic Period, 1.5-to-2.5-year-old female individual.

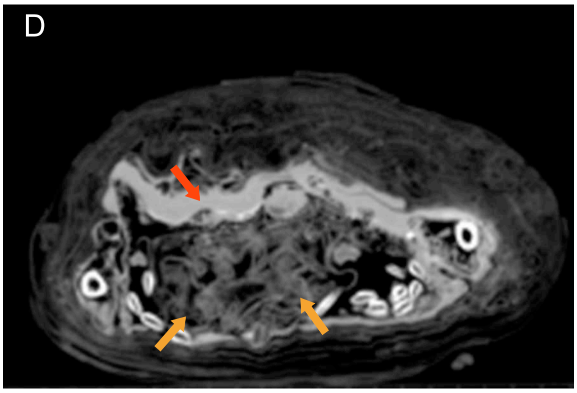

(A) Scattered dark-colored areas on the textiles were caused by the use of embalming fluids between the textiles. Note a thin layer of gauze was added in modern times during conservation for protection. The female's name is written in Greek characters on one of the outermost bandages across the torso (© SMB - Ägyptisches Museum und Papyrussammlung, photo: S. Steiß). **(B) Coronal plane** - Skull and torso are connected by thin longitudinal foreign objects (most probably palm panicles) (red arrows). **(C) Sagittal plane** - Segments of the longitudinal foreign objects (red arrows) are visible on the severely flattened torso as well as resin-like embalming substances (orange arrows). **(D) Axial plane** - Textiles (orange arrows) are present inside the eviscerated torso, covered by embalming substances (red arrow) (CT image reconstructions: © German Mummy Project, S. Zesch).

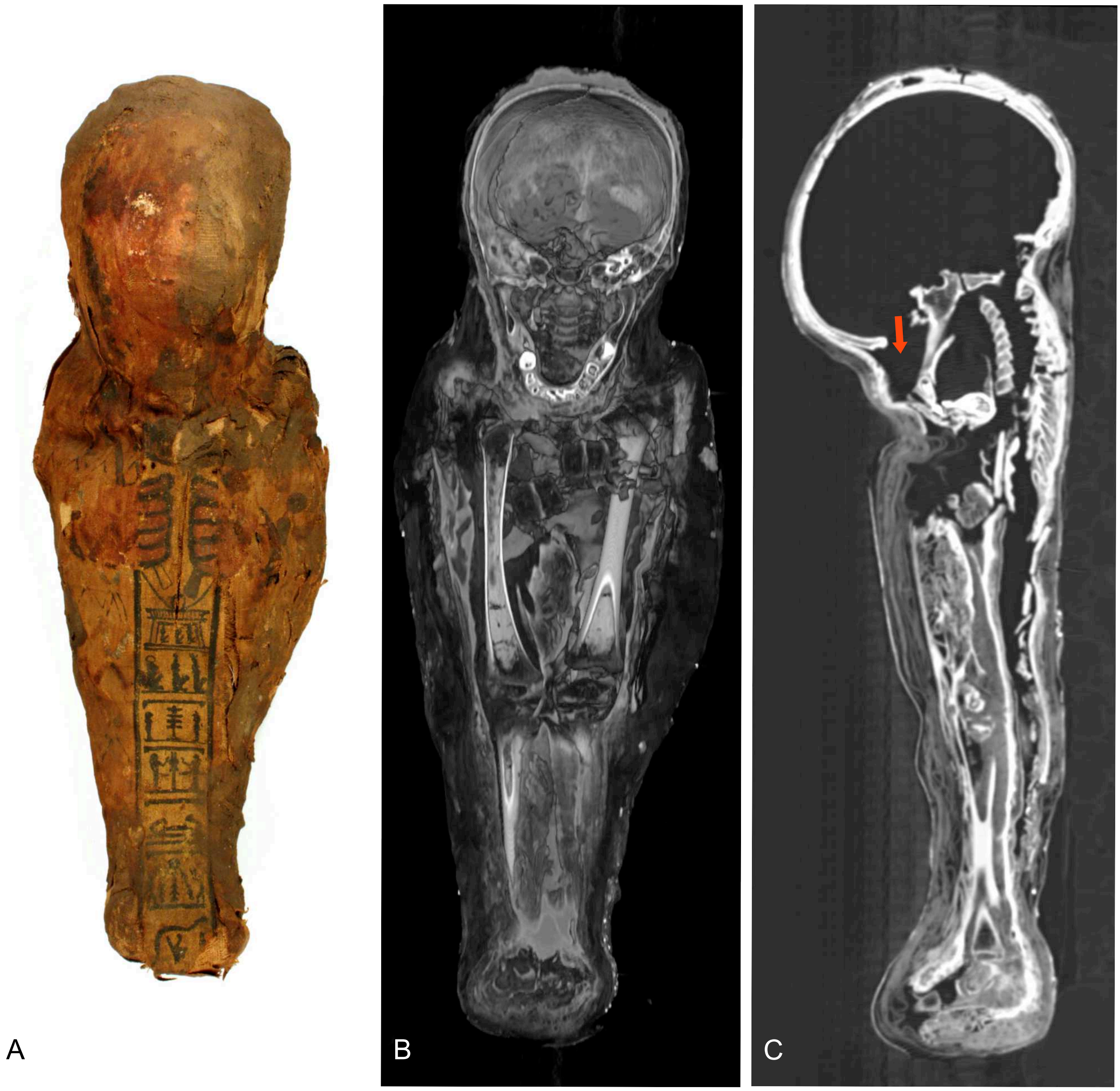

Case 4 (III 8226) - Unknown archaeological site, Ptolemaic Period to Roman Period, 2-to-3-year-old individual of unknown sex.

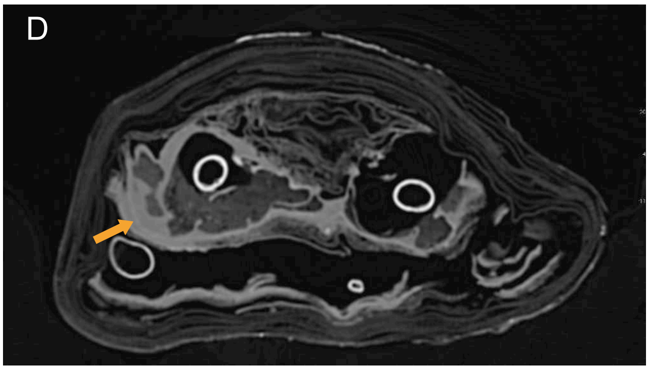

(A) Remnants of mummy decoration, painted on the outermost textile layer, are preserved (© German Mummy Project, photo: W. Rosendahl). **(B) Coronal plane -** The femurs were placed at the level of the shoulders by the embalmers indicating that the vertebrae, ribs and pelvic bones were absent during mummification. **(C) Sagittal plane** - The brain was removed through the ethmoid (red arrow). Due to massive damage to the the thoracic and abdominal areas, internal organs were not preserved. **(D) Axial plane** - Resin-like embalming substances (orange arrows) are visible at the level of the shoulders with the dislocated leg bones included in the image (CT image reconstructions: © German Mummy Project, S. Zesch).

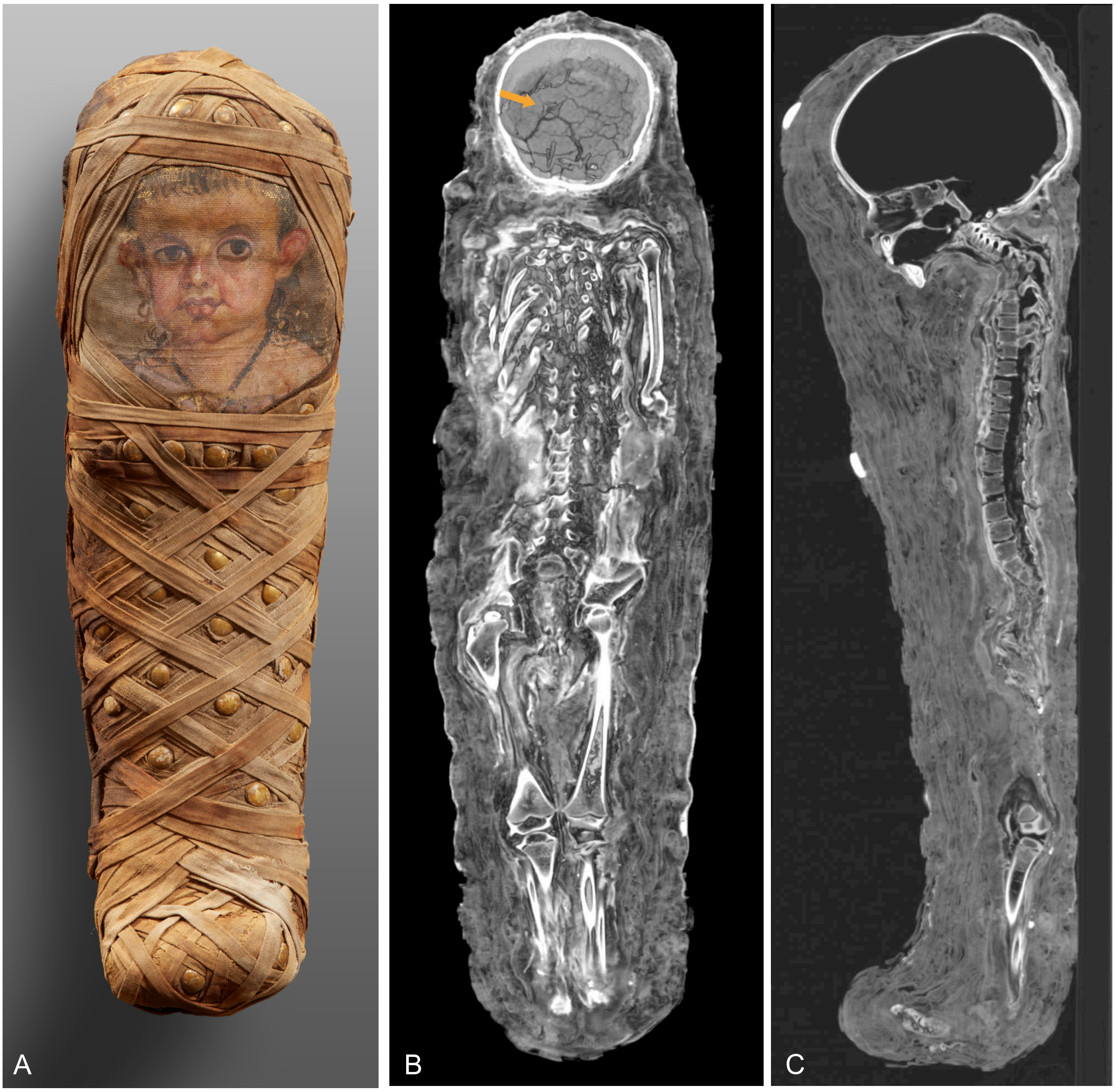

Case 5 (ÄM 11413) - Hawara, Roman Period, 2-to-3-year-old male individual.

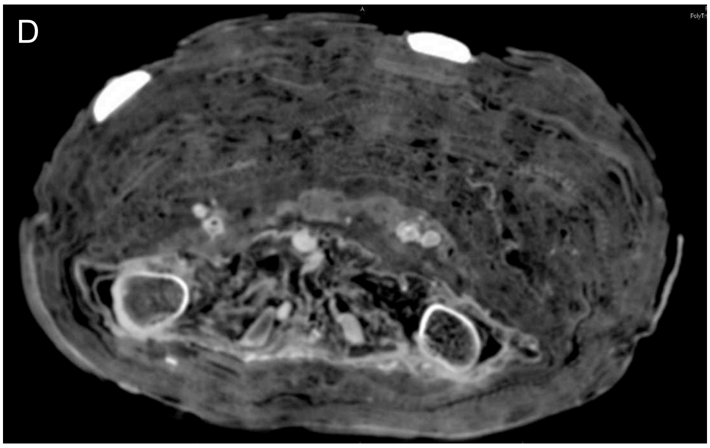

(A) The outermost textile layers were created by cross-folded bandages in a rhomboid pattern and decorated with gilded stucco buttons. A mummy portrait was painted on the mummy shroud below the outermost bandages (© SMB - Ägyptisches Museum und Papyrussammlung, photo: S. Steiß). **(B) Coronal plane** - Remnants of the shrunken brain are preserved inside the skull (orange arrow). Several bones (for example ribs, vertebrae, and pelvic bones) are disarticulated and dislocated within the torso. **(C) Sagittal plane** - Internal organs were not identified. Due to the extensively flattened torso, it remains unknown if these had been removed artificially. **(D) Axial plane** - The mummy was wrapped by tightly packed layers of textiles. Resin-like embalming materials were not applied (CT image reconstructions: © German Mummy Project, S. Zesch).

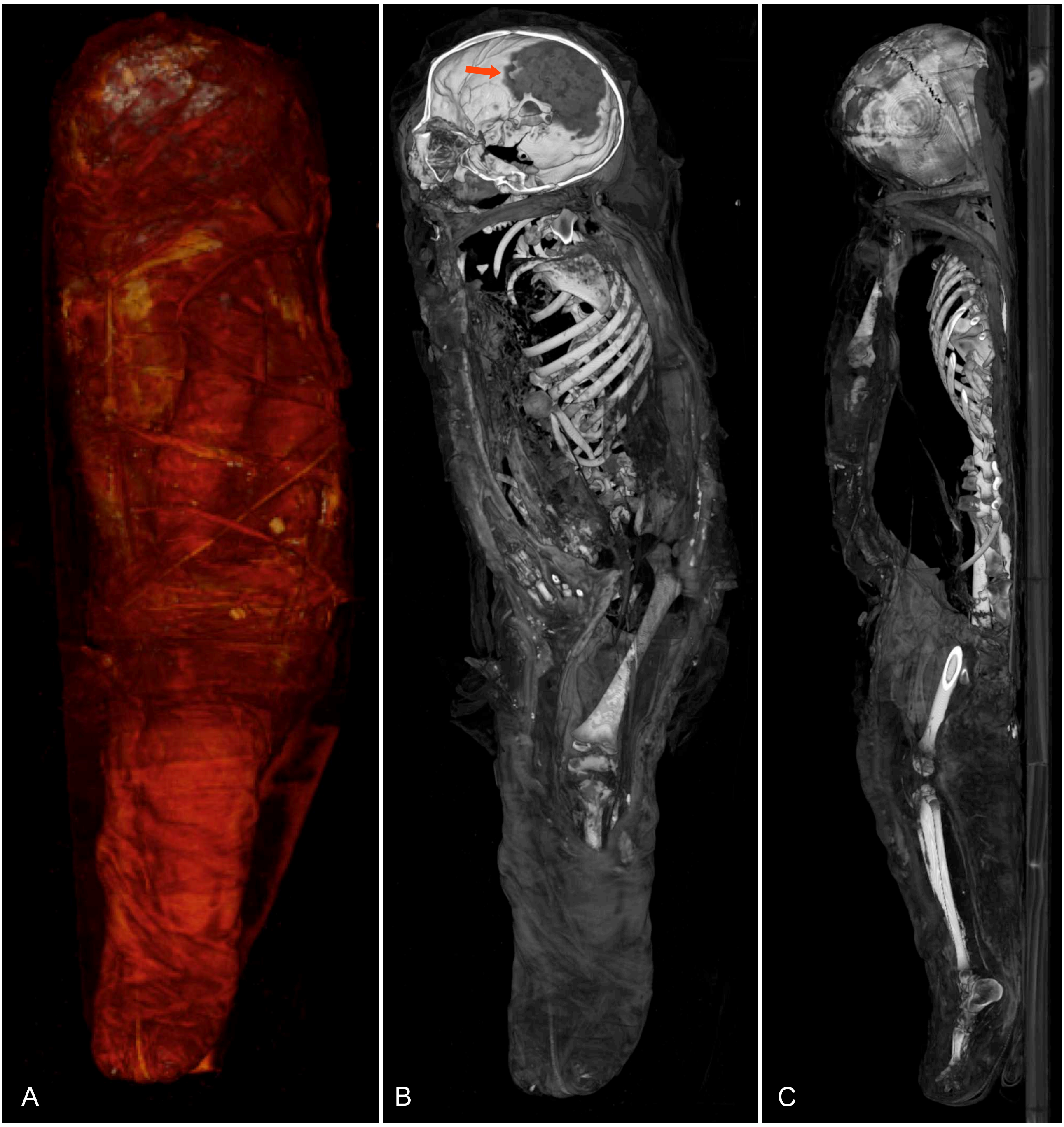

Case 6 (PM 6356) - Asyut (?), Old Kingdom to First Intermediate Period, 2-to-4-year-old individual of unknown sex.

(A) 3D-Volume rendered reconstruction illustrates the mummy with the arms individually wrapped below the outermost textiles. **(B) Coronal plane** - Soft tissues are poorly preserved and internal organs were not identified. The brain (red arrow) was not removed. **(C) Sagittal plane** - Note the flattened torso inside the wrappings. Bones are broadly disarticulated and dislocated. **(D) Axial plane** - Arms and hands (orange arrows) were individually wrapped before the body was covered by textiles (CT image reconstructions: © German Mummy Project, S. Zesch).

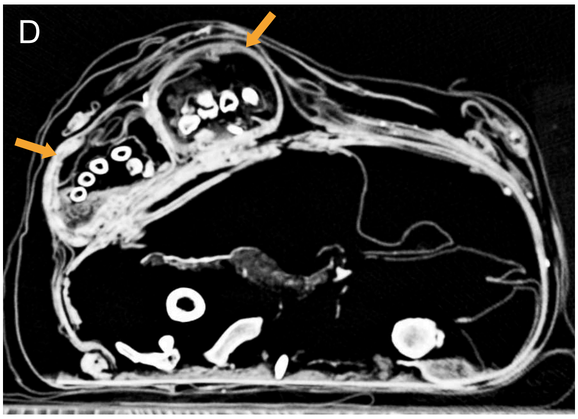

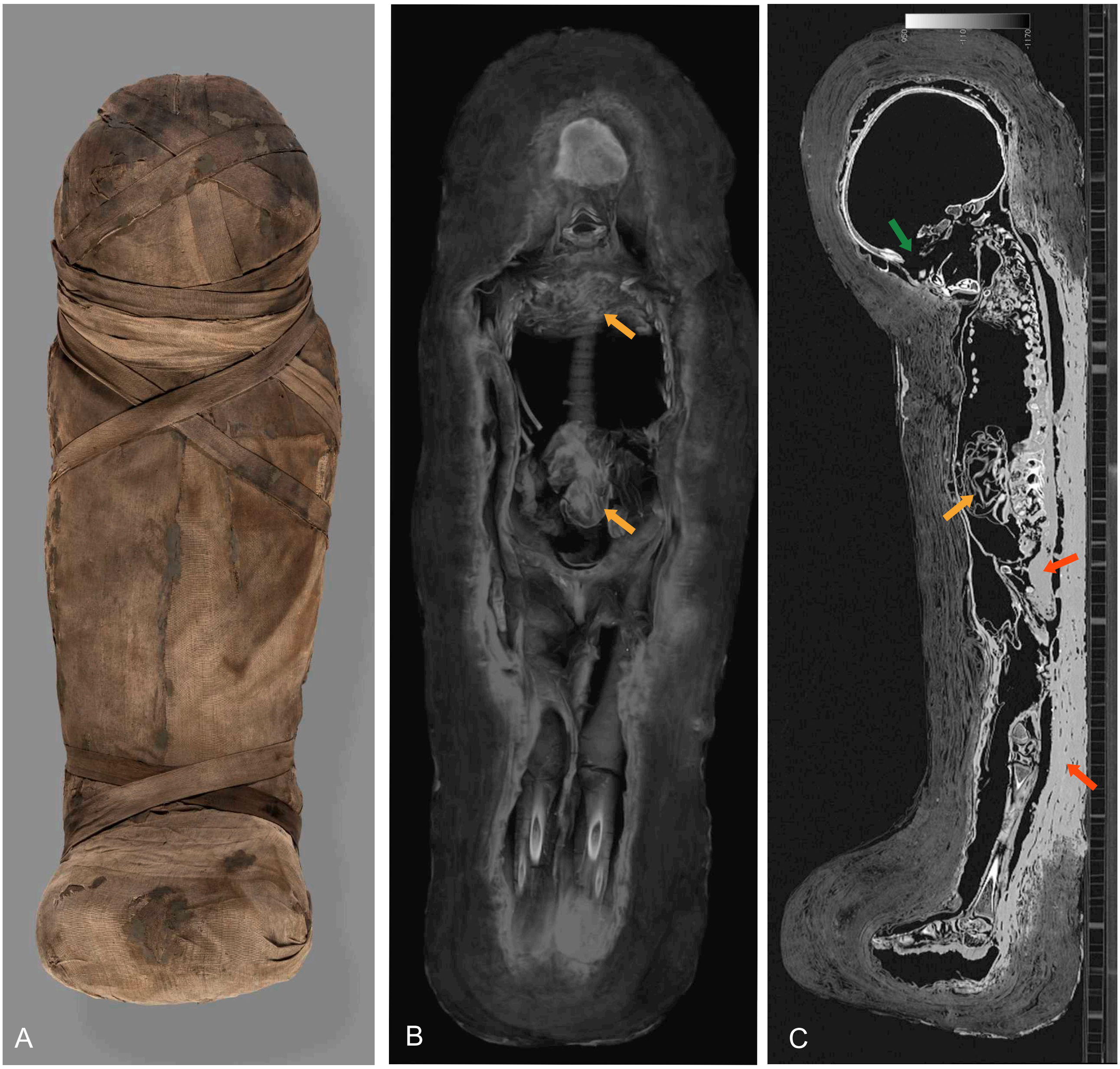

Case 7 (ÄM 505/03) - Western Thebes, Roman Period, 2.5-to-4-year-old male individual.

(A) Scattered areas of dark-colored textiles indicate the use of embalming substances between the wrappings (© SMB - Ägyptisches Museum und Papyrussammlung, photo: S. Steiß). **(B) Coronal plane** – Loosely folded textile bundles inside the eviscerated body are present at the levels of the shoulders and abdomen (orange arrows). **(C) Sagittal plane** - The brain was removed through the ethmoid (green arrow). Loosely folded textiles are visible inside the abdominal cavity (orange arrow). The body, wrapped by densely packed layers of textiles, was extensively treated with resin-like embalming materials (red arrows). **(D) Axial plane** - Note textile fillings (orange arrow) and resin-like embalming materials (red arrow) inside the body (CT image reconstructions: © German Mummy Project, S. Zesch).

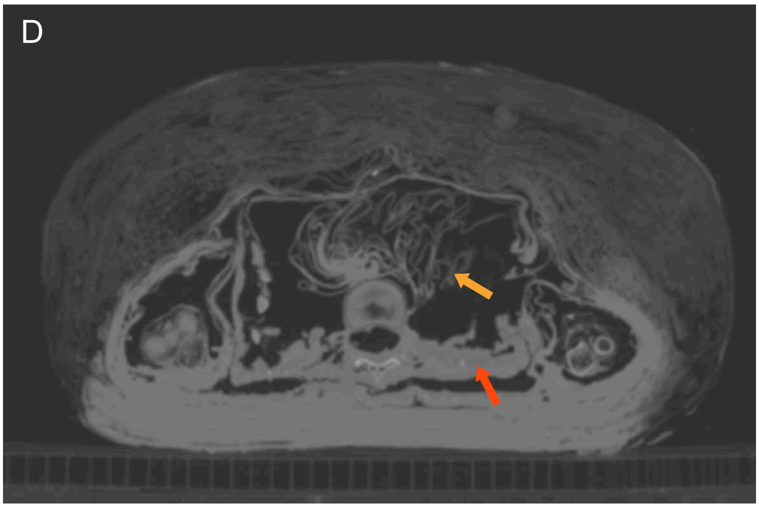

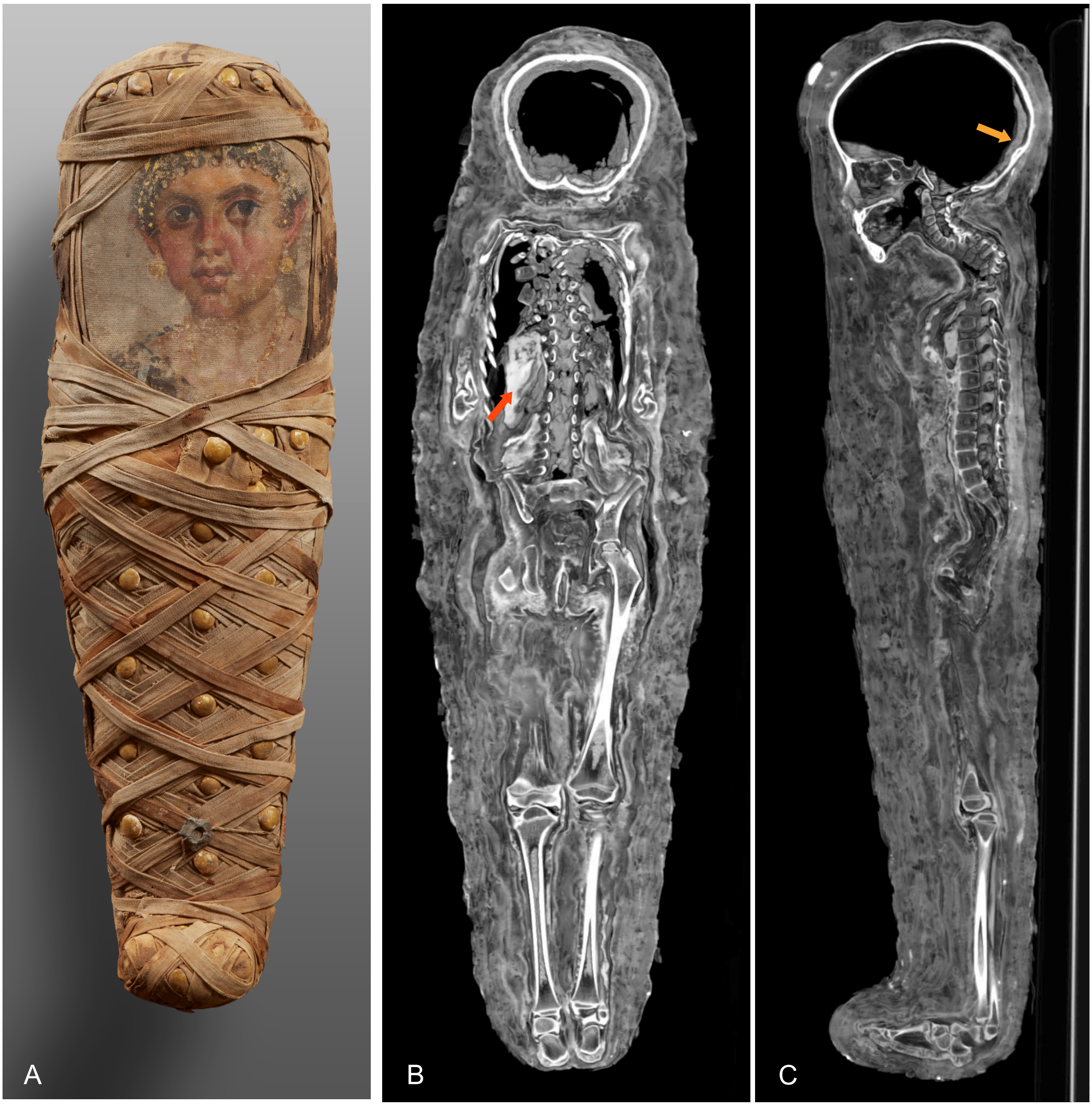

Case 8 (ÄM 11412) - Hawara, Roman Period, 2.5-to-4-year-old female individual.

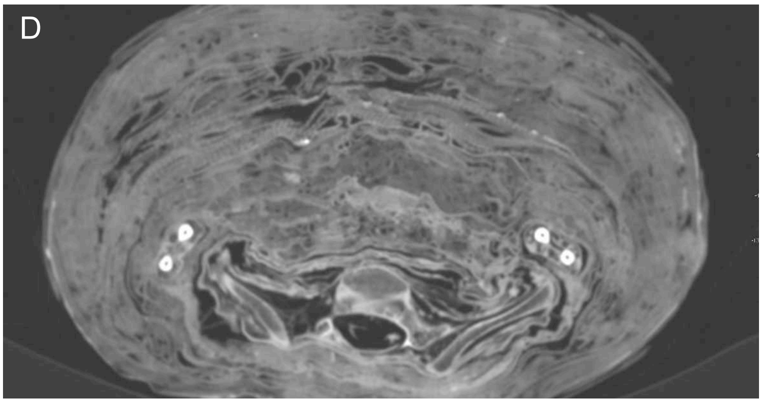

(A) The outermost textile layers were created by cross-folded bandages in a rhomboid pattern. The mummy was decorated with gilded stucco buttons and a mummy portrait painted on a shroud below the outermost bandages (© SMB - Ägyptisches Museum und Papyrussammlung, photo: S. Steiß). **(B) Coronal plane** - Remnants of the liver (red arrow) are visible inside the torso. **(C) Sagittal plane** - The brain is present as a shrunken mass inside the skull (orange arrow). The mummy lacks any evidence indicating the use of resin-like embalming materials. **(D) Axial plane** - The remarkably flatten body was wrapped by densely packed layers of textile (CT image reconstructions: © German Mummy Project, S. Zesch).

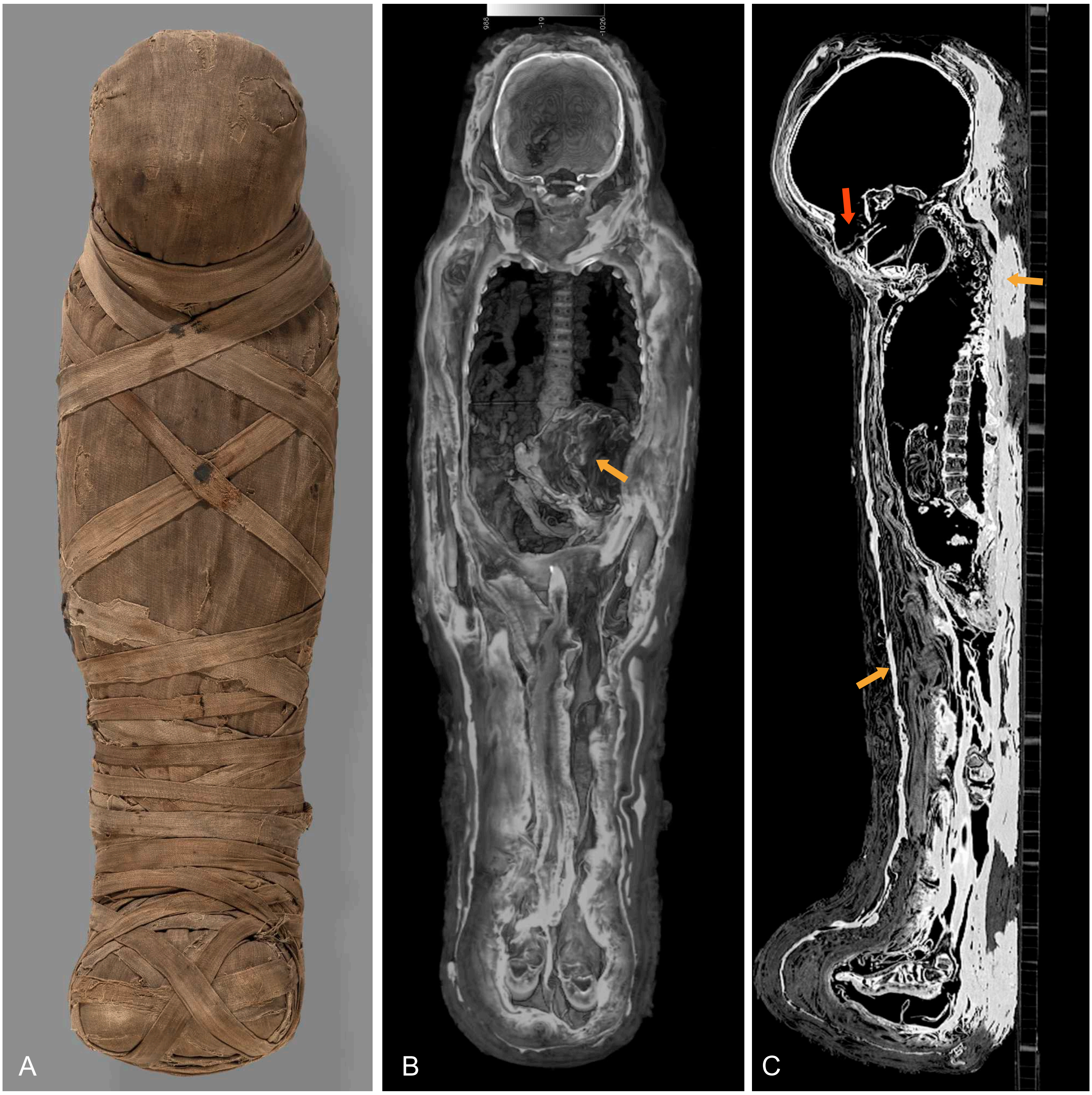

Case 9 (ÄM 504/03) - Western Thebes, Roman Period, 3-to-4-year-old female individual.

(A) The mummy was wrapped by sheets of textile and bandages in transverse and cross-shaped pattern (© SPK, SMB - Ägyptisches Museum und Papyrussammlung, photo: A. Paasch). **(B) Coronal plane -** Loosely folded textiles (orange arrow) are visible at the level of the abdominal cavity. **(C) Sagittal plane -** The brain was removed through the ethmoid (red arrow). The eviscerated body was extensively treated with resin-like embalming materials (orange arrows) **(D) Axial plane -** Note a left-sided abdominal incision (orange arrows) used for both evisceration and insertion of a bundle of textiles (CT image reconstructions: © German Mummy Project, S. Zesch).

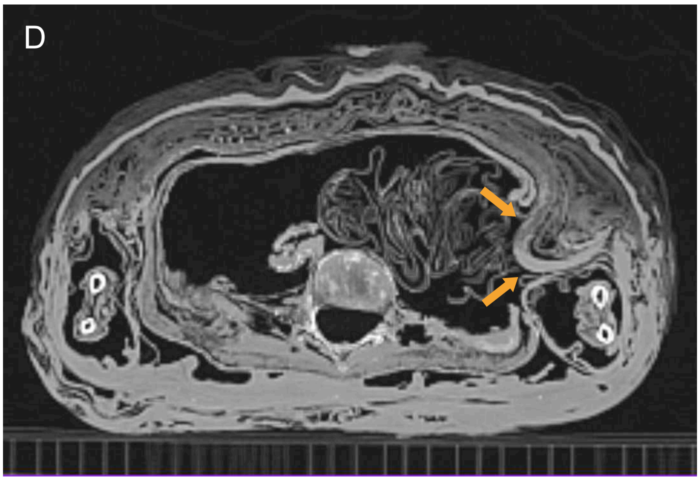

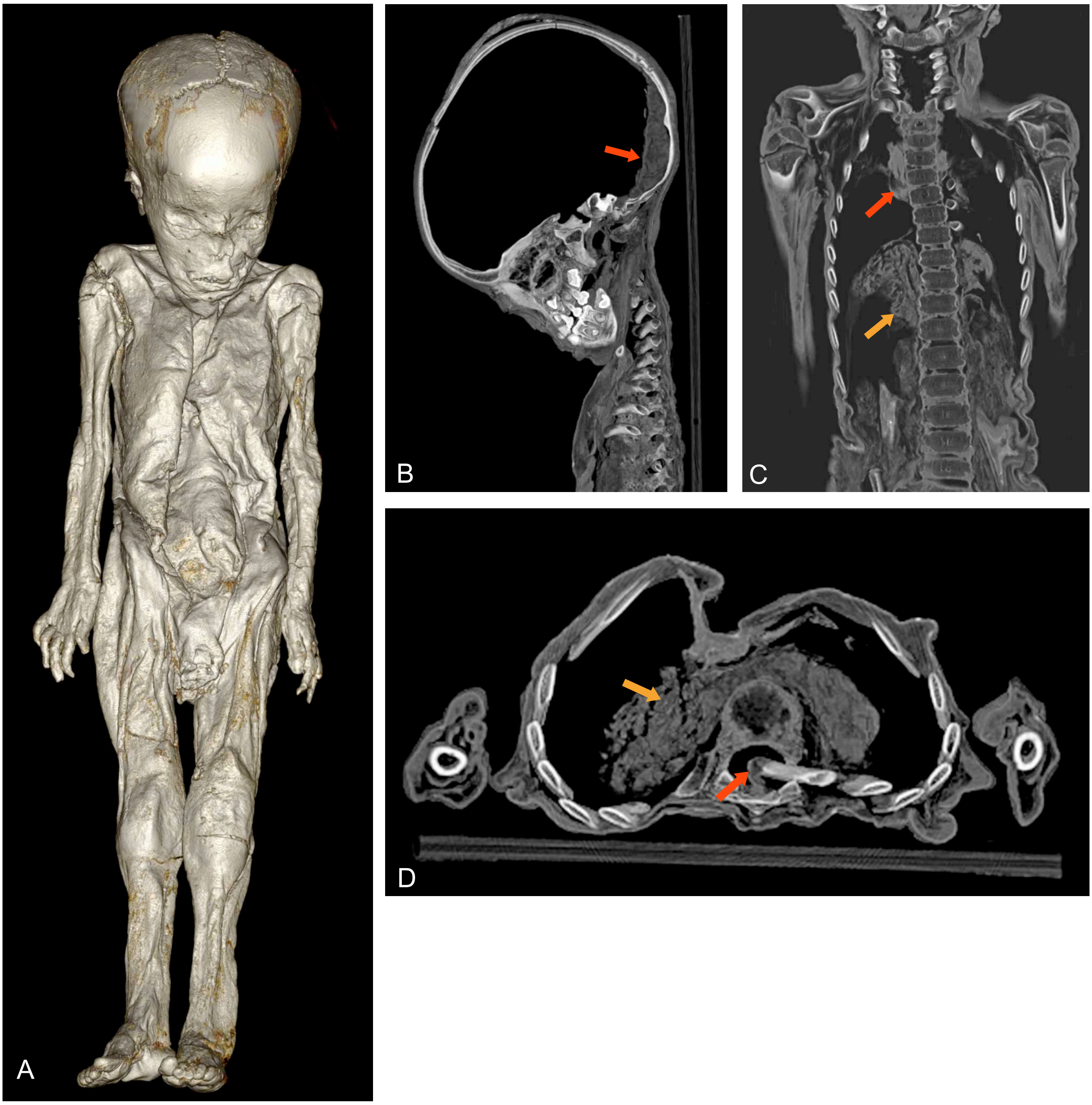

Case 10 (6027) - Unknown archaeological site, Roman Period, 3-to-4-year-old male individual.

(A) 3D-Volume rendered reconstruction illustrates the unwrapped body with the central longitudinal segment of the torso sunken dorsally. **(B) Sagittal plane** - The brain was identified as a shrunken mass inside the skull (red arrow). The chin is flexed onto the upper chest. **(C) Coronal plane** - Note remnants of the desiccated heart (red arrow), and the liver (orange arrow). **(D) Axial plane** - The disarticulated vertebral end of the left 10th rib (red arrow) is dislocated into the spinal canal. Note also remnants of the liver (orange arrow) (CT image reconstructions: © German Mummy Project, S. Zesch).

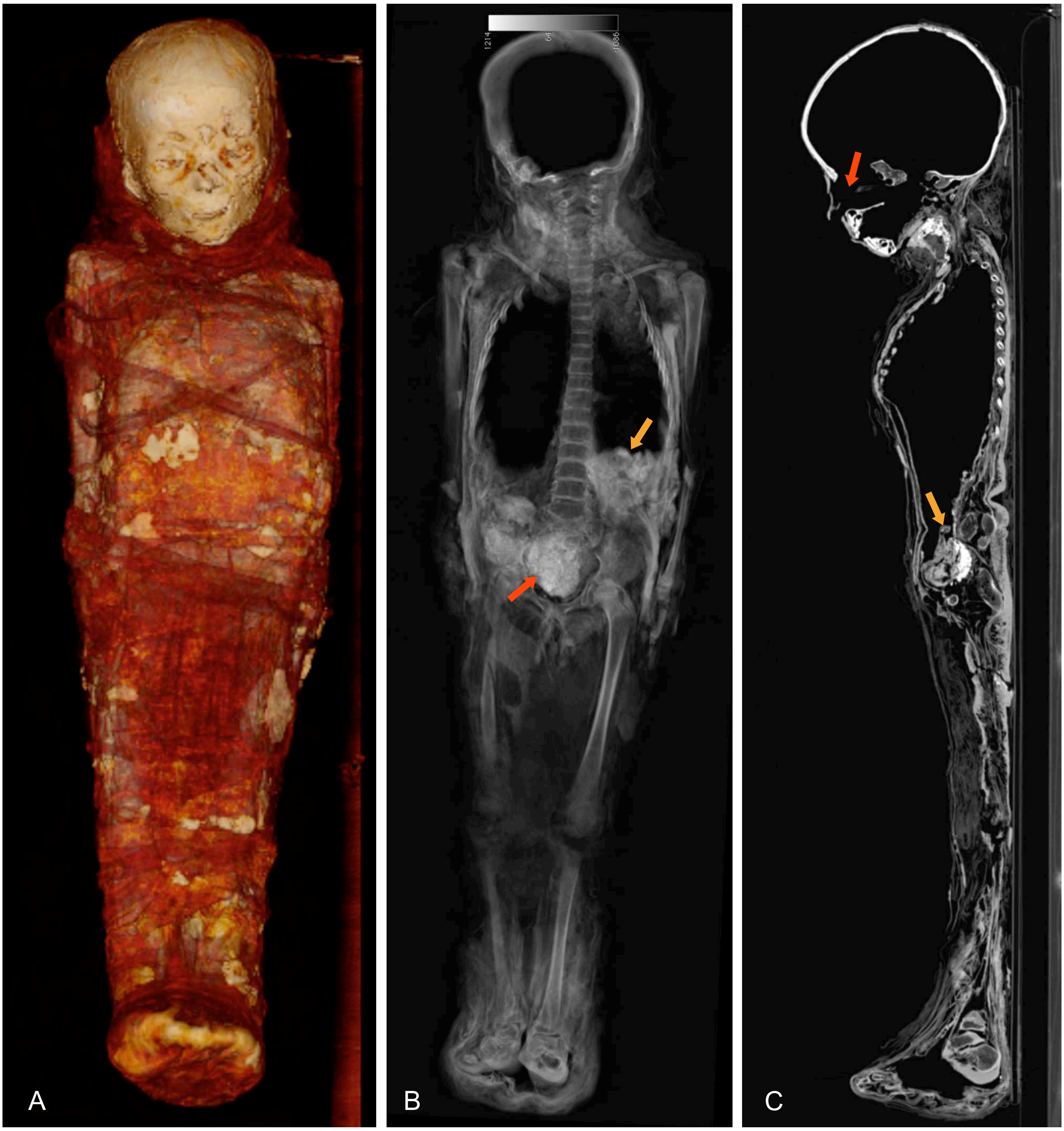

Case 11 (PM 5206) - Unknown archaeological site, Roman Period, 3-to-4-year-old male individual.

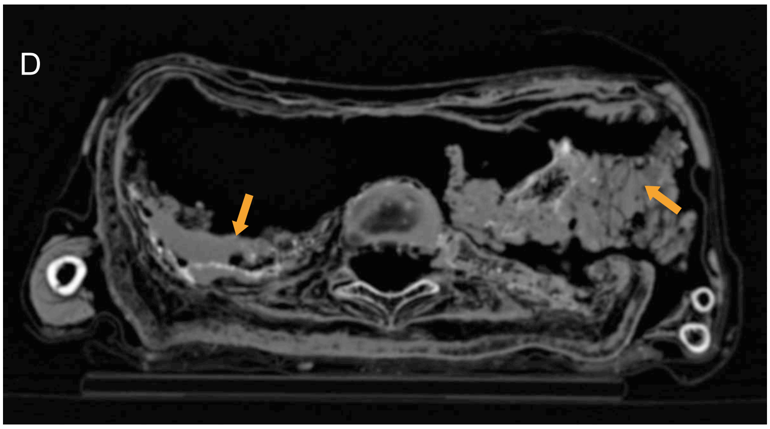

(A) 3D-Volume rendered reconstruction illustrates the mummy in textiles with the head unwrapped. **(B) Coronal plane** - Textile filling is visible inside the lower left thoracic region (orange arrow). A mixture of granular materials was inserted into the pelvis (red arrow). **(C) Sagittal plane** - Note the removal of brain through the ethmoid (red arrow), the eviscerated torso, and mummification materials inside pelvis (orange arrow). **(D) Axial plane** - Resin-like embalming substances (orange arrows) are visible at the abdominal level (CT image reconstructions: © German Mummy Project, S. Zesch).

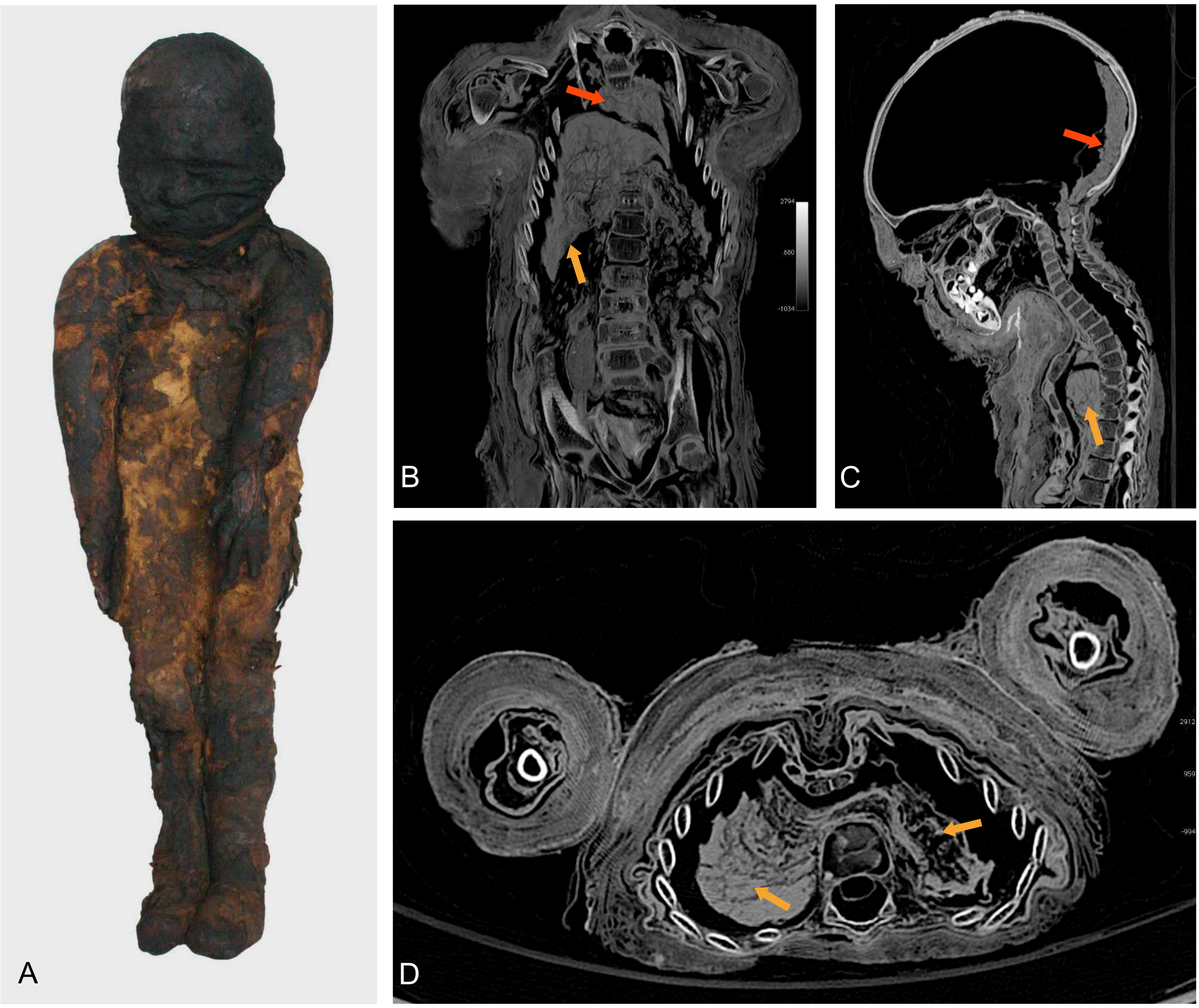

Case 12 (ÄS 18) - Unknown archaeological site, Late Period to early Ptolemaic Period, 3-to-4-year-old male individual.

(A) The head, arms, fingers and legs are individually wrapped with textile in tightly packed layers. The body, however, is incompletely covered, likely due to post-preservation removal of some wrapping material. Dark-colored areas of textiles indicate the application of embalming substances between the various textile layers (© German Mummy Project, photo: S. Zesch) **(B) Coronal plane** - The reconstruction illustrates the non-eviscerated body with a focus on the heart (red arrow), displaced into the upper part of the chest, as well as on the liver (orange arrow), partially migrated into the chest cavity. **(C) Sagittal plane** - Radiopaque embalming substances with granular components are visible between the textiles under the chin. Shrunken brain remnants (red arrow) are preserved inside the skull and within the cervical spinal canal. Also note the remnants of the dislocated liver (orange arrow) inside the chest cavity. **(D) Axial plane** - Parts of the dislocated liver (orange arrows) are illustrated within the thoracic cavity. Note the densely packed layers of textiles covering the upper arms and the torso (CT image reconstructions: © German Mummy Project, S. Zesch).

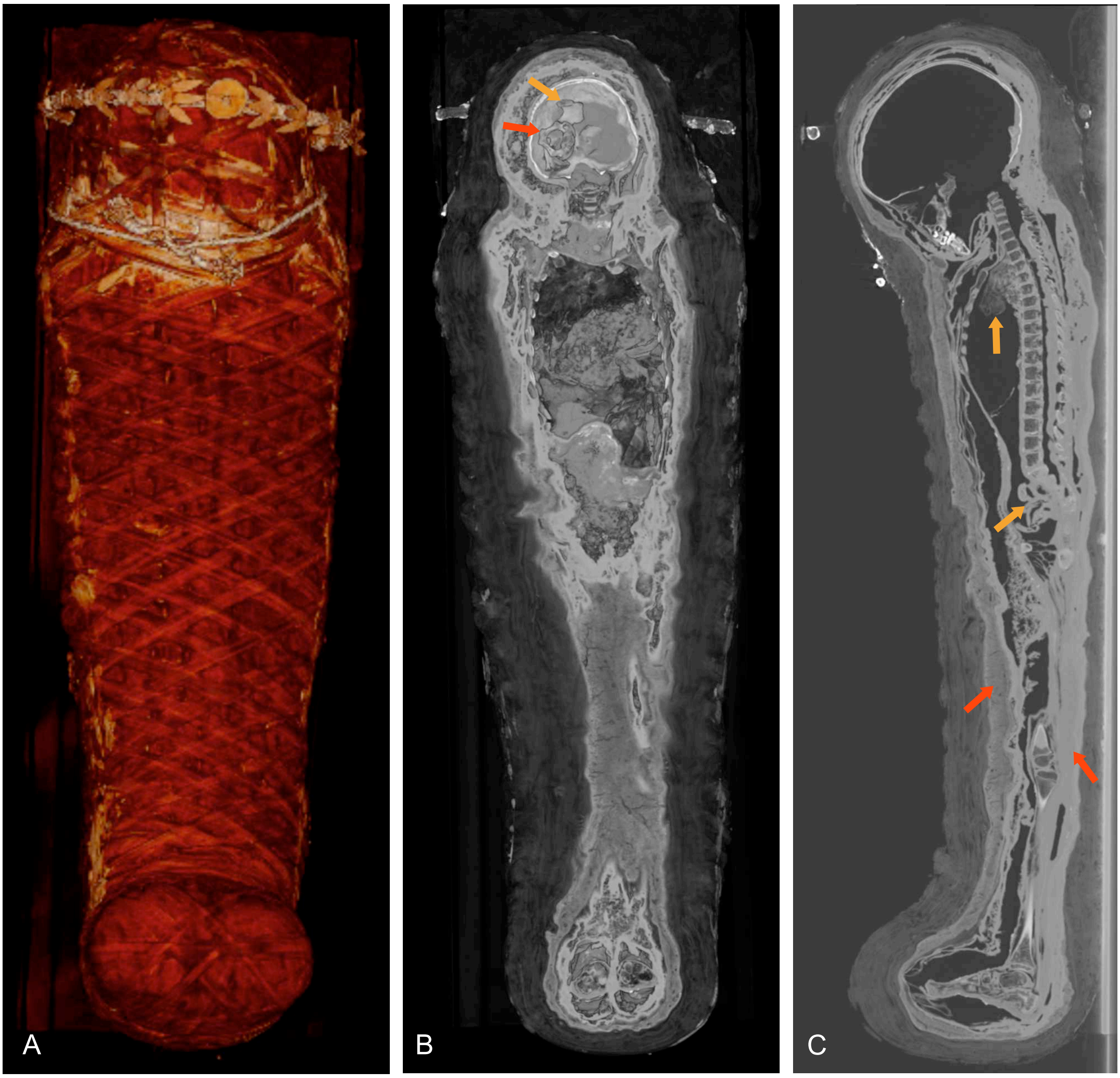

Case 13 (Cat. 2230/1) - Western Thebes, Roman Period, 4-to-5-year-old male individual.

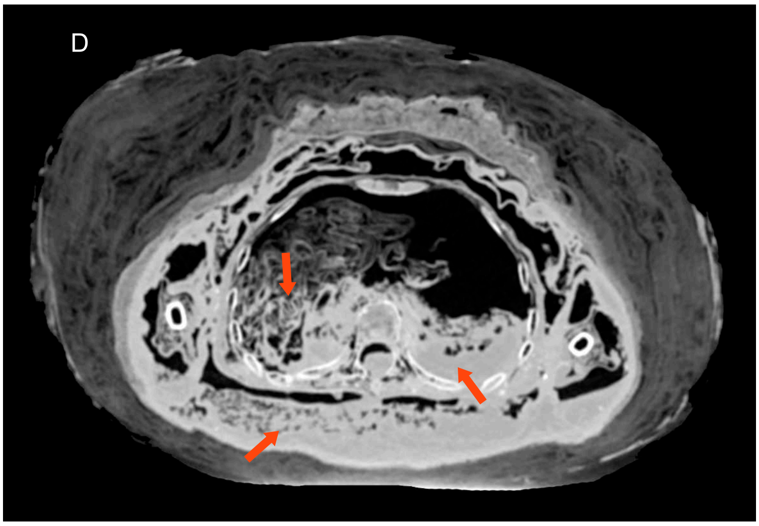

(A) The outermost textile layers were created by cross-folded bandages in a rhomboid pattern. A wreath was placed on the head. **(B) Coronal plane** - Below the outermost textile layers, the body was densely wrapped by various sheets of textile. The pars basilaris of the occipital bone (orange arrow) and the second cervical vertebrae (red arrow) are dislocated inside the cranial cavity indicating brain removal via the cranio-cervical route. **(C) Sagittal plane** - Note the presence of few loosely folded textiles impregnated with resin-like substances (orange arrows) within the upper thorax and the abdominal cavity of the eviscerated body. Resin-like embalming substances were extensively applied to the body and its wrappings (red arrows) **(D) Axial plane** - Note the leakage of resin-like embalming substances into bones, soft tissues, and textiles (red arrows) (CT image reconstructions: © German Mummy Project, S. Zesch).

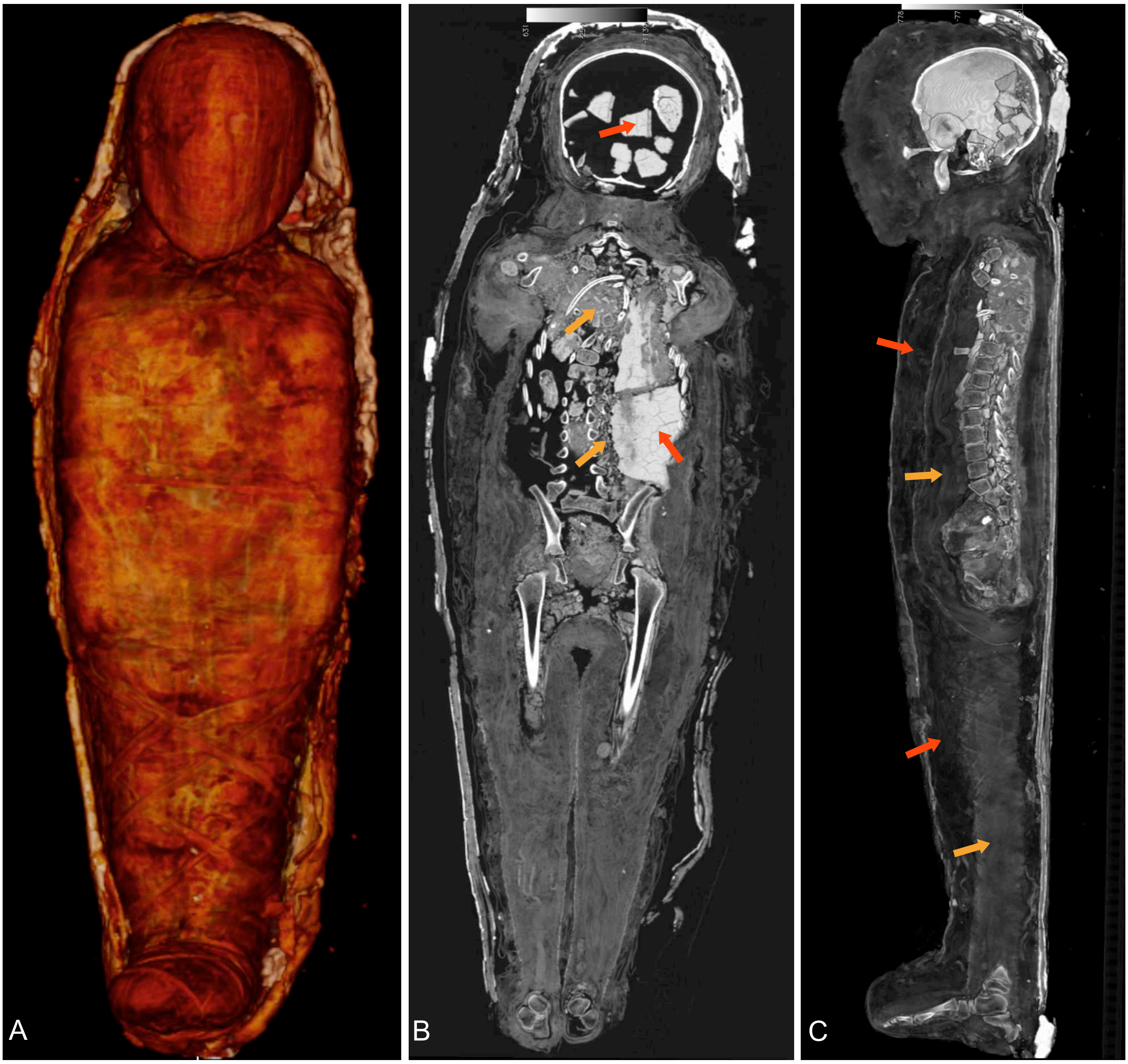

Case 14 (ÄM 16800/03) - Abusir el-Meleq, Roman Period, 4-to-6-year-old female individual.

(A) The mummy, inside a cartonnage coffin, was wrapped in a cocoon-like style created by densely packed layers of textile sheets and bandages. **(B) Coronal plane** - Note the presence of resin-like embalming substances inside the excerebrated skull and eviscerated torso (red arrows), as well as several granular materials within the torso (orange arrows). **(C) Sagittal plane** - The head, torso and limbs have been wrapped individually (orange arrows) before the whole body was completely wrapped by sheets of textiles (red arrows). **(D) Axial plane** - Note the arms and torso individually wrapped (red arrows) as well as textiles present within the pelvis/ abdomen (orange arrow) (CT image reconstructions: © German Mummy Project, S. Zesch).

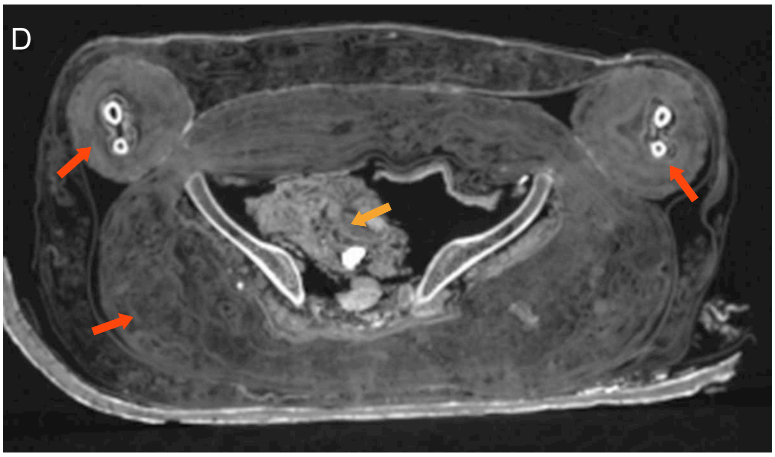

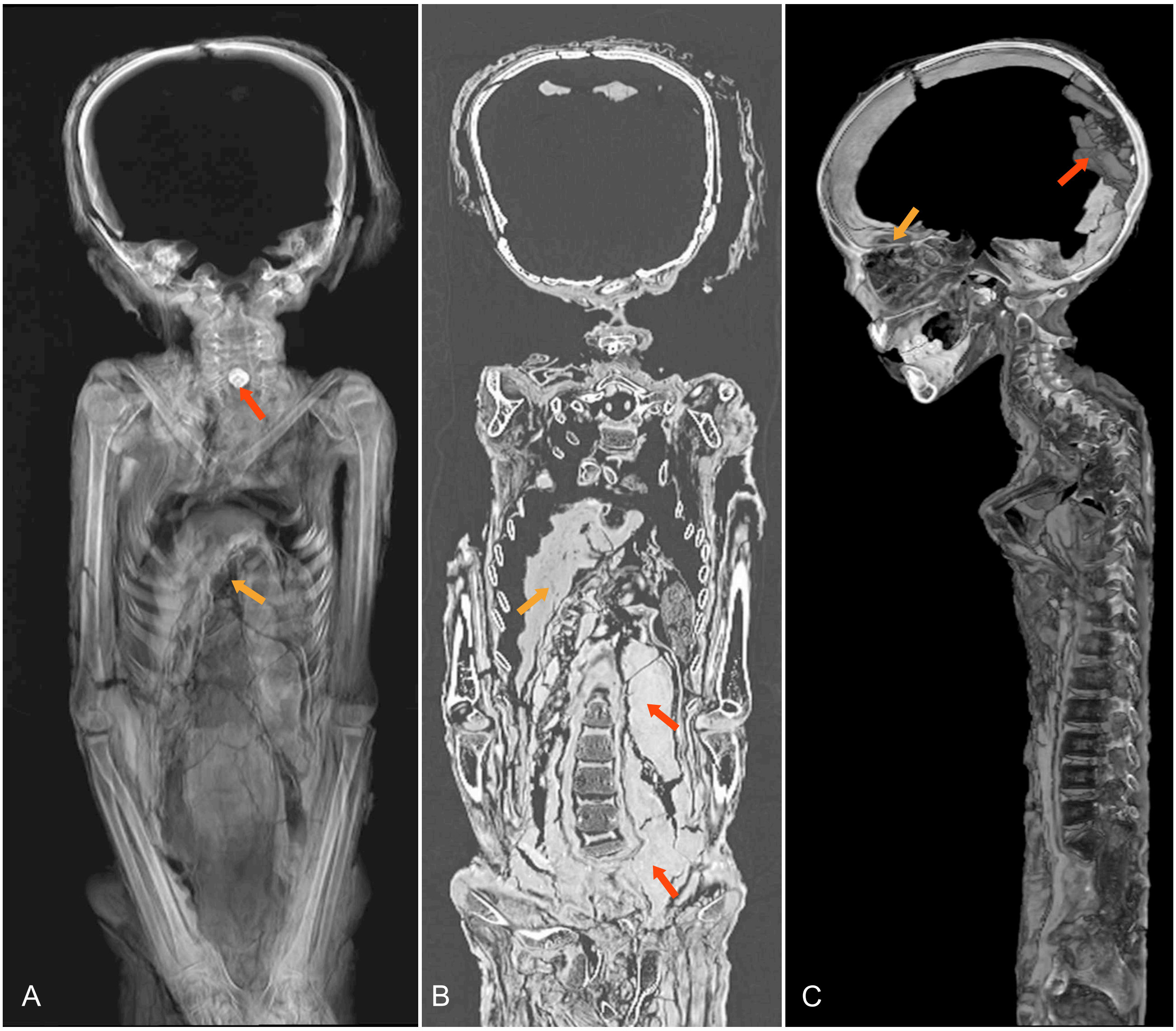

Case 15 (ÄM 723) - Western Thebes, Third Intermediate Period to Late Period, 5-to-6-year-old male individual.

(A) Coronal plane - The central segment of the thoracic and abdominal area is sunken dorsally (orange arrow). Note an isolated molar (red arrow), dislocated onto the level of the 5th cervical vertebra. **(B) Coronal plane** - Several bones of the non-eviscerated mummy are disarticulated and fragmented. Note the presence of the liver (orange arrow) dislocated into the chest cavity. Resin-like embalming substances (red arrows) are visible on the skin at the thoracic and abdominal levels. **(C) Sagittal plane** - The brain was removed via the ethmoid route (orange arrow). Pieces of resin-like embalming substances (red arrow) were identified inside the cranial cavity. Note the gibbus deformity at the cervico-thoracic junction, marked pectus carinatum deformity of the sternum and flatten torso of this individual (CT image reconstructions: © German Mummy Project, S. Zesch).

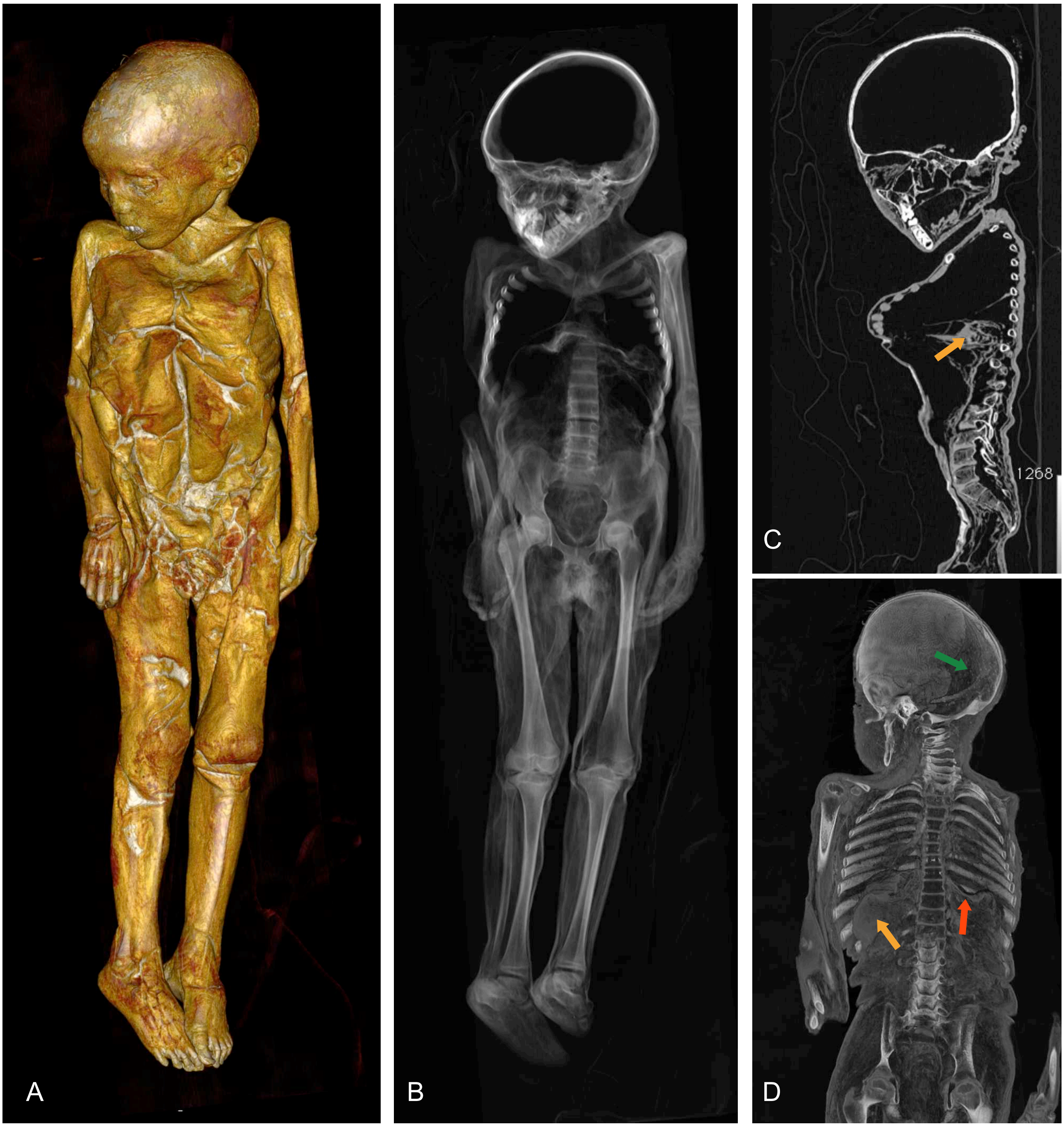

Case 16 (Ä 16) - Unknown archaeological site, assumed Ptolemaic Period to Roman Period, 5-to-7-year-old male individual.

(A) 3D-Volume rendered reconstruction illustrates the unwrapped body with the central longitudinal segment of the thorax and abdomen sunken dorsally. **(B) Coronal plane** - Thick-slab mean intensity multi-planar reconstruction illustrates the well-preserved skeleton as well as few remnants of internal organs. **C) Sagittal plane** - Remnants of the desiccated heart (orange arrow) are visible within thoracic cavity. Note the remarkable flatten abdominal region. **(D) Coronal plane** - Note few remnants of the desiccated brain (green arrow), the liver (orange arrow), and the diaphragm (red arrow) within the body cavities (CT image reconstructions: © German Mummy Project, S. Zesch).

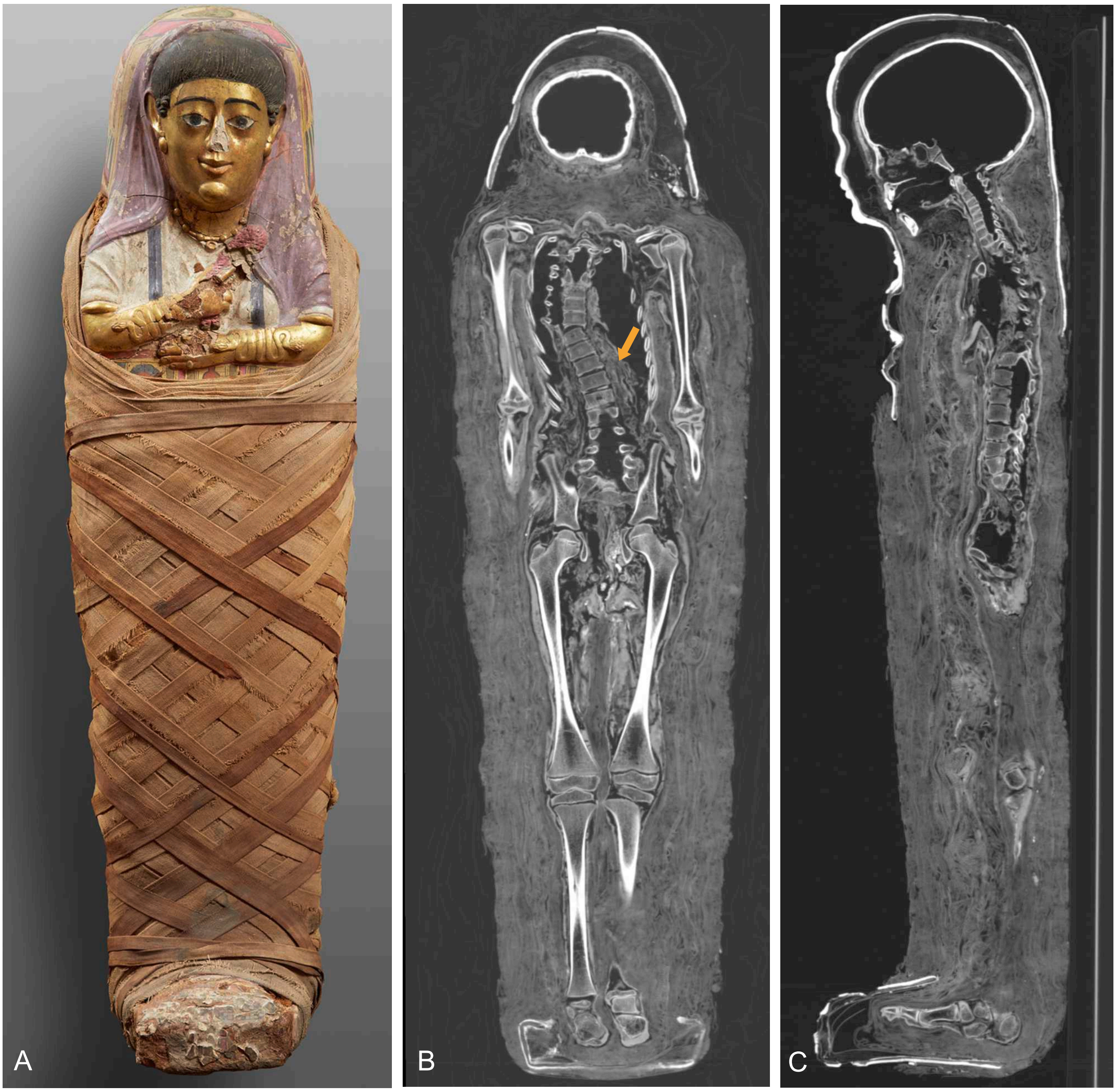

Case 17 (ÄM 12125) - Hawara, Roman Period, 6-to-7-year-old female individual.

(A) The outermost textile layers were created by cross-folded bandages in a rhomboid pattern. The mummy is decorated with a painted and gilded mummy mask (© SMB - Ägyptisches Museum und Papyrussammlung, photo: S. Steiß). **(B) Coronal plane** - Remnants of the desiccated internal organs (orange arrow) are visible inside the torso. Note the disarticulated thoracic column. **(C) Sagittal plane** - Note the absence of the brain as well as the remarkable flatten shape of the torso. **(D) Axial plane** - The desiccated body was wrapped by many densely packed layers of textiles. **(E) Sagittal plane** - The brain was removed through the ethmoid route (red arrow) (CT image reconstructions: © German Mummy Project, S. Zesch).

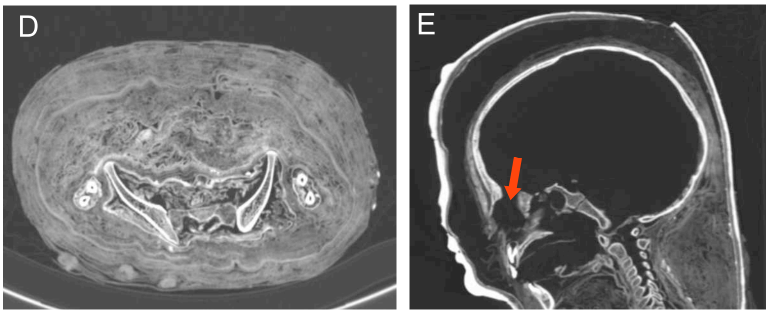

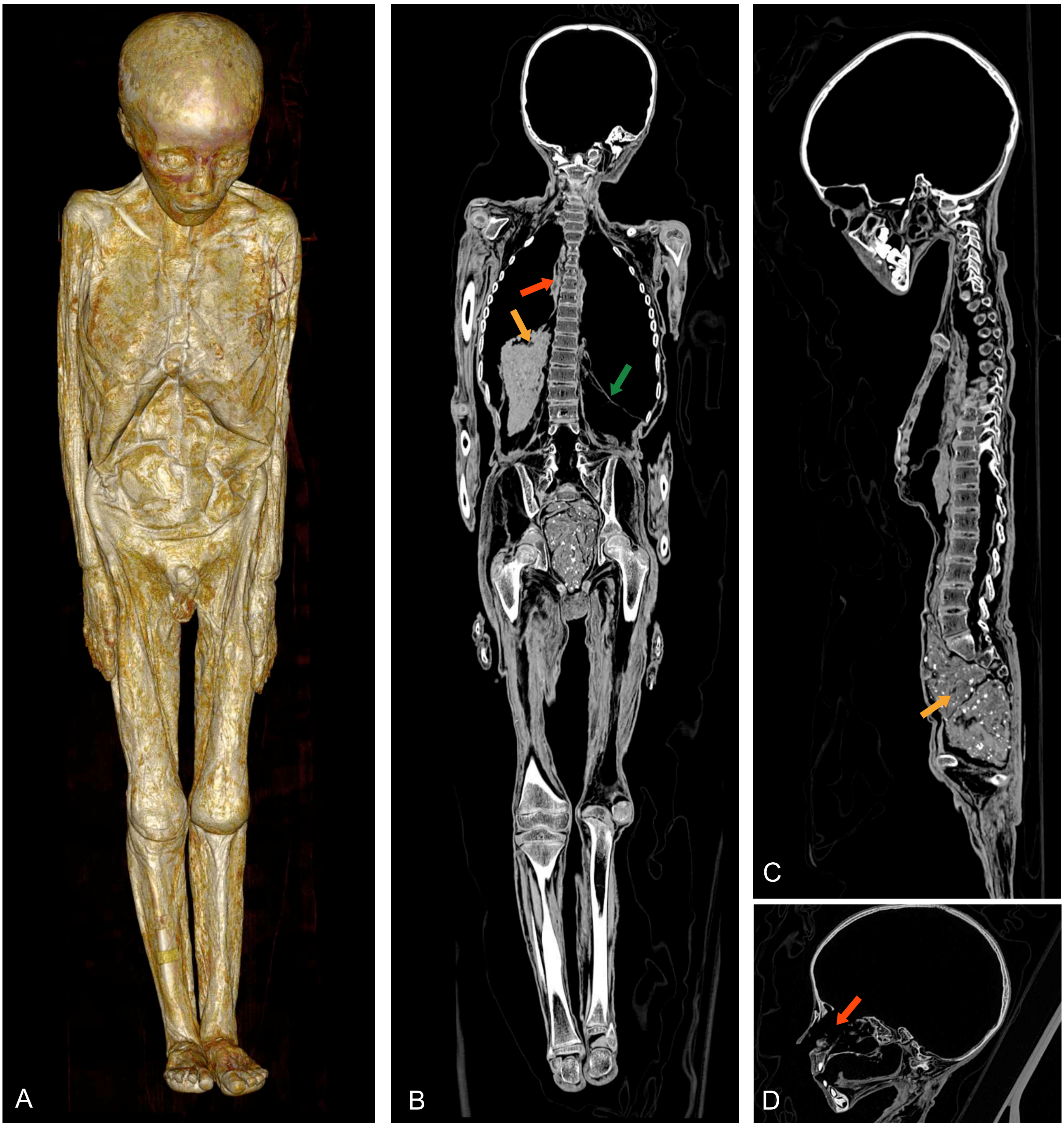

Case 18 (Ä 17) - Unknown archaeological site, assumed Ptolemaic Period to Roman Period, 7-to-9-year-old male individual.

(A) 3D-Volume rendered reconstruction illustrates the unwrapped body with the sunken abdominal skin. **(B) Coronal plane -** The desiccated heart (red arrow), liver (orange arrow), and diaphragm (green arrow) are preserved. **(C) Sagittal plane -** Remnants of the desiccated internal organs are visible within the chest cavity, as well as filling material (orange arrow) made of textiles and granular material within the abdominal and pelvis regions **(D) Sagittal plane** - The brain was removed through the ethmoid route (red arrow). **(E) Axial plane -** A bundle of textiles and granular materials (orange arrow), most probably inserted through the perineal route, is visible within the pelvis (CT image reconstructions: © German Mummy Project, S. Zesch).

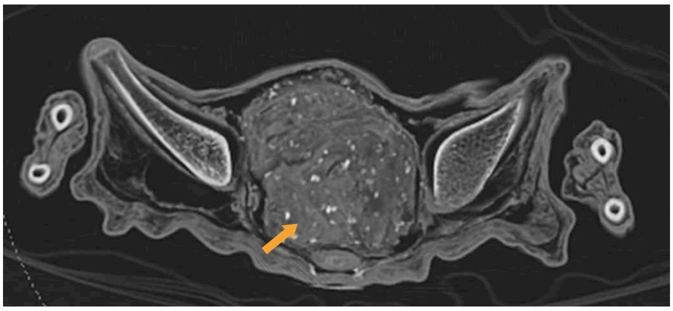

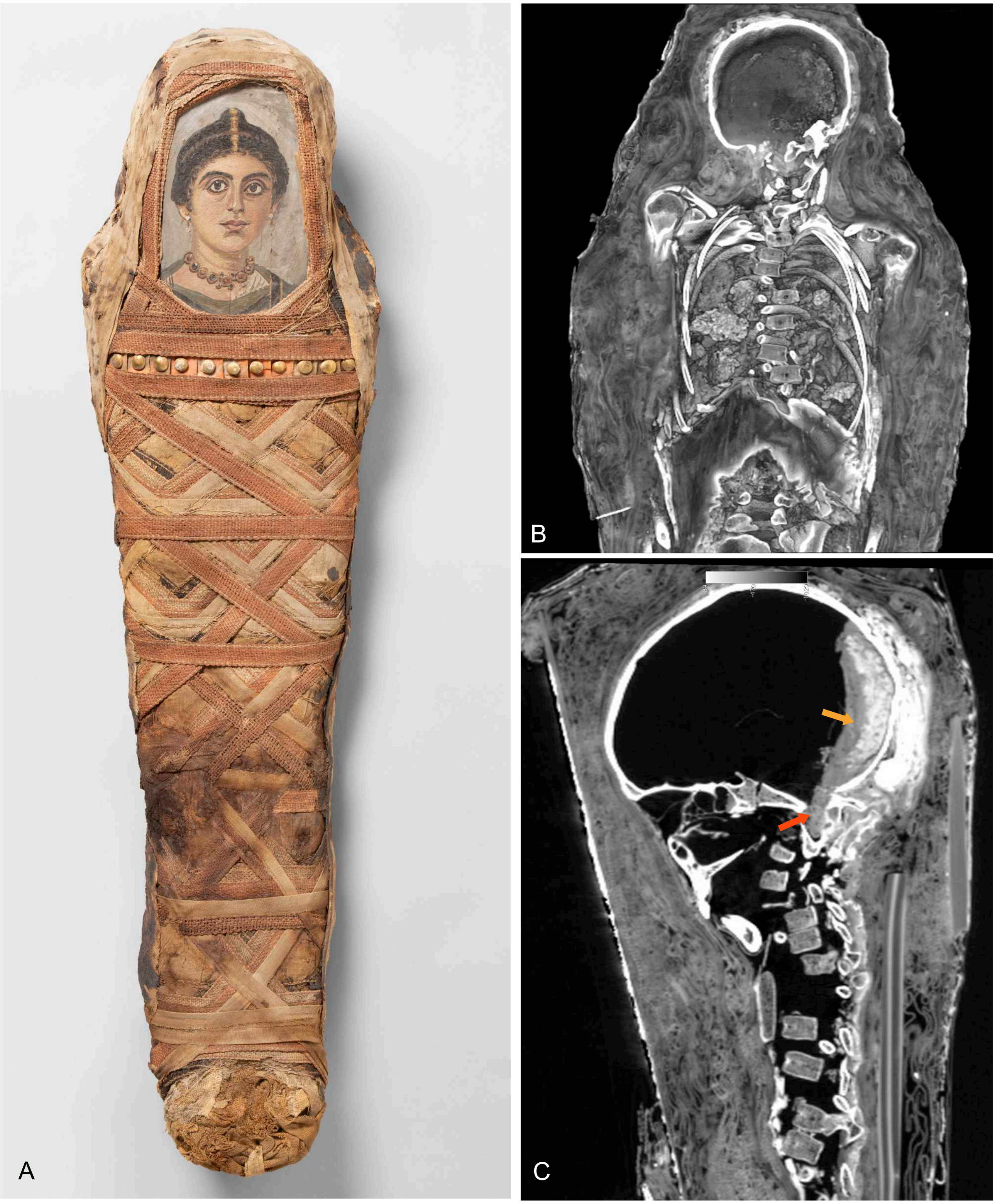

Case 19 (ÄM 36101/01) - Er-Rubayat, Roman Period, 9-to-10-year-old female individual.

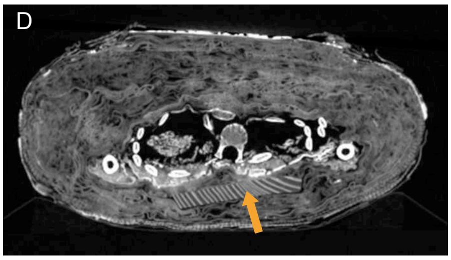

(A) The outermost textile layers were created by cross-shaped bandages, decorated with gilded stucco buttons, and a mummy portrait painted on a wooden panel (© SMB - Ägyptisches Museum und Papyrussammlung, photo: S. Steiß). **(B) Coronal plane** - The ribs and vertebrae are disarticulated and dislocated. **(C) Sagittal plane** - The brain was incompletely removed through the cranio-cervical route (red arrow), and radio-dense embalming substances (orange arrow) were inserted inside the skull. **(D) Axial plane** - The non-eviscerated body, lying on a wooden board (orange arrow), was densely wrapped by sheets of textiles (CT image reconstructions: © German Mummy Project, S. Zesch).

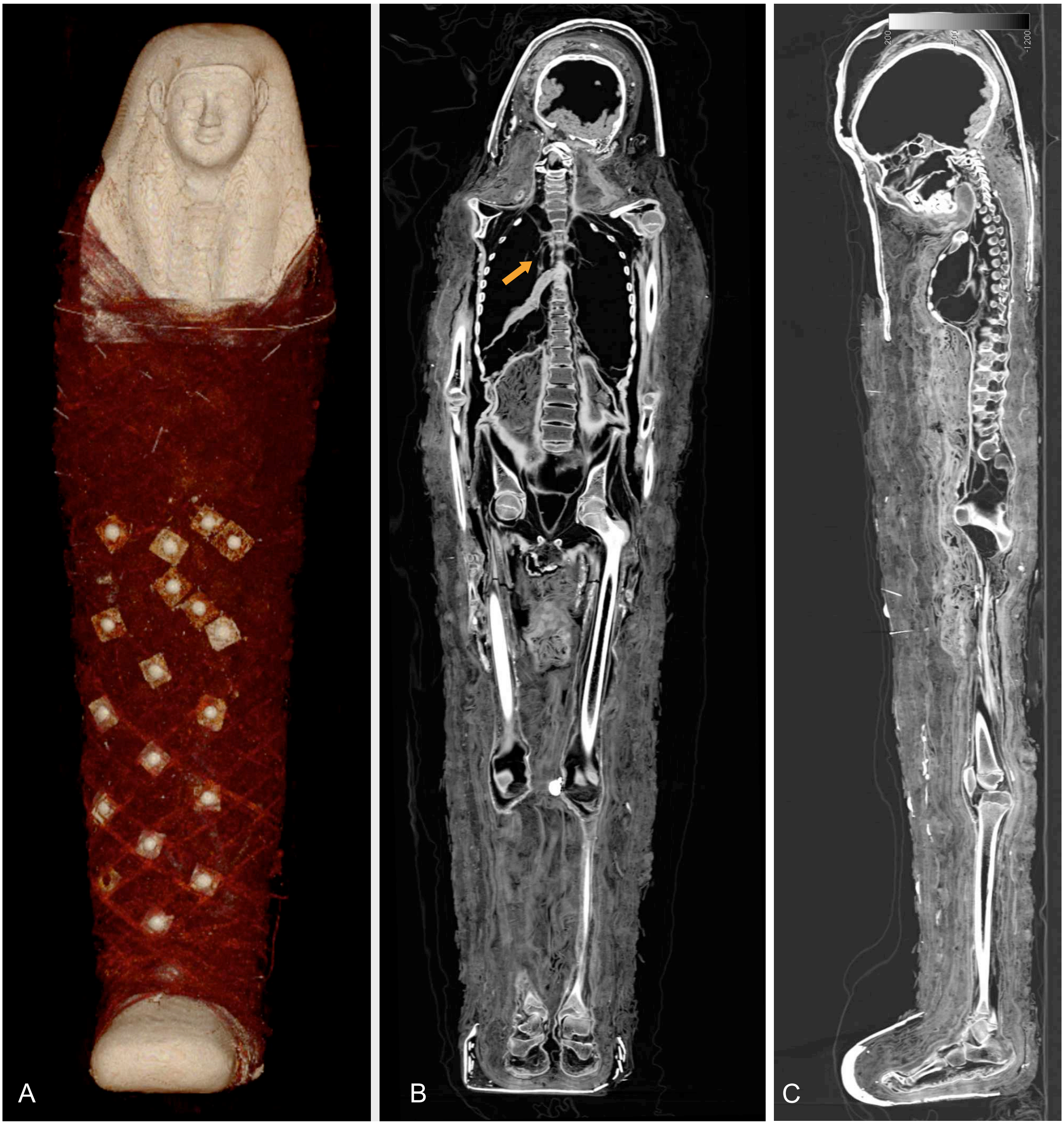

Case 20 (Ä 15) - Unknown archaeological site, assumed Ptolemaic Period to Roman Period, 9-to-10-year-old male individual.

(A) 3D-Volume rendered reconstruction illustrates the mummy was wrapped by cross-folded bandages with stucco buttons, and covered by cartonnage trappings, including a mask and foot cartonnage. **(B) Coronal plane -** Remnants of desiccated lung tissue (orange arrow) are visible within the thoracic cavity of the non-eviscerated mummy. **(C) Sagittal plane** - The brain was not removed. Note the flatten contour of the abdominal and pelvis regions. **(D) Axial plane** - The body was wrapped by densely packed textiles. Note a segment of the cartonnage mask (orange arrow) between the textiles at the level of the thorax (CT image reconstructions: © German Mummy Project, S. Zesch).

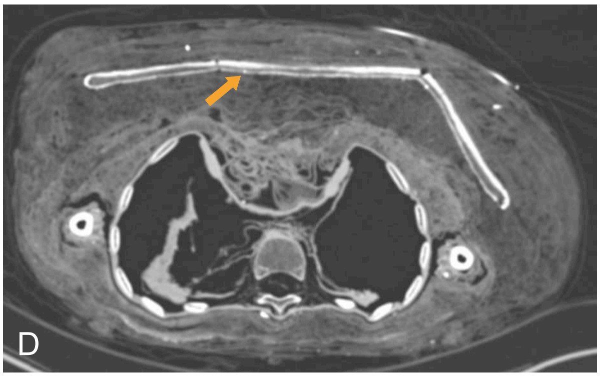

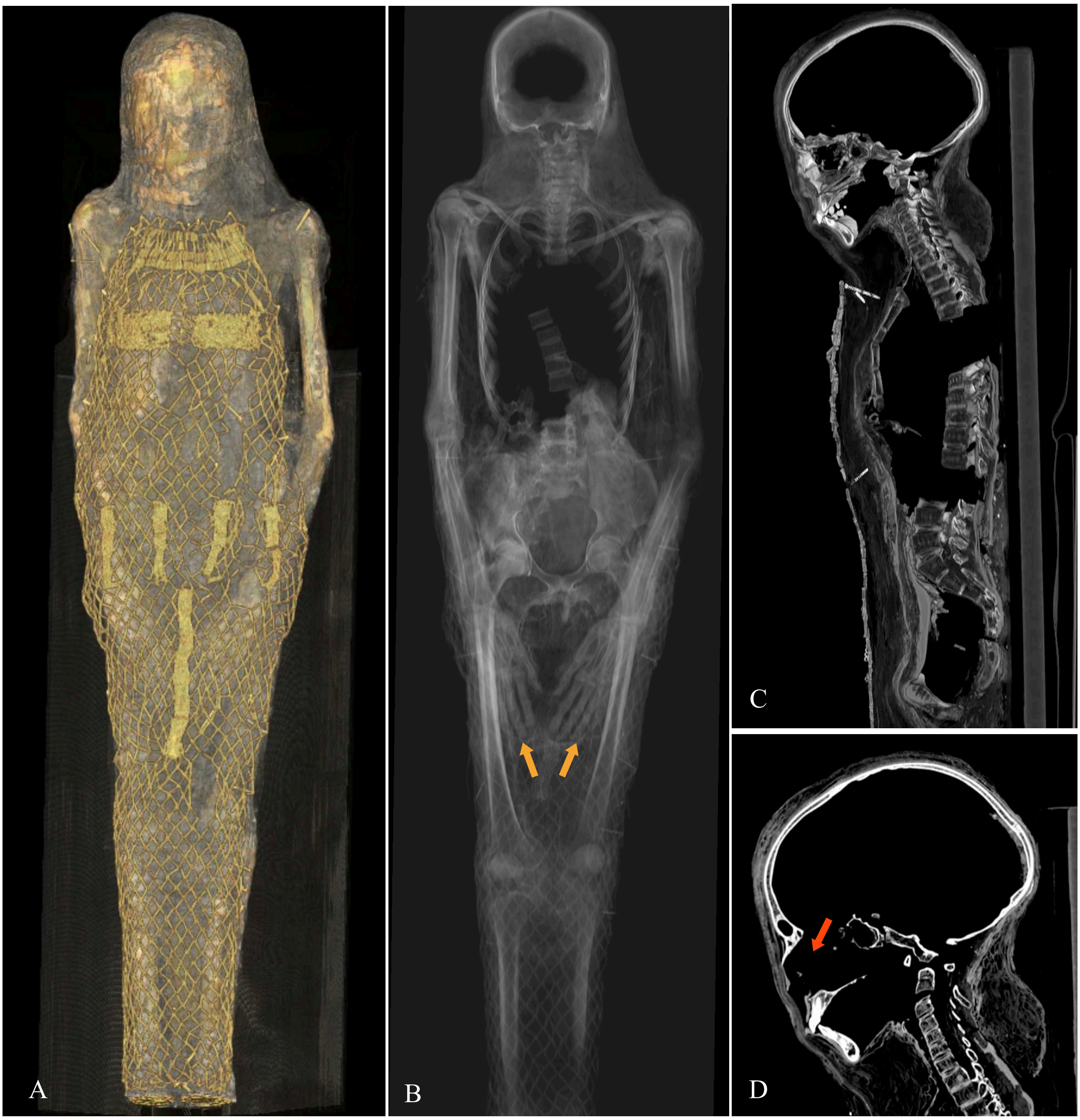

Case 21 (III 30) - Western Thebes, Late Period to early Ptolemaic Period, 12-to-14-year-old male individual.

(A) 3D-Volume rendered reconstruction illustrates the mummy in textile wrappings, covered by a net made of faience beads. **(B) Coronal plane** - The hands (orange arrows) were placed on the tights. **(C) Sagittal plane** - Inner organs were removed during mummification. Parts of the vertebral column were disarticulated and dislocated within the torso, presumably during anatomical manipulation in modern times. **(D) Sagittal plane** - The brain was removed through the ethmoid route (red arrow). **(E) Axial plane** - Resin-like embalming substances (red arrows) are visible as a thin layer on the abdomen (CT image reconstructions: © German Mummy Project, S. Zesch).

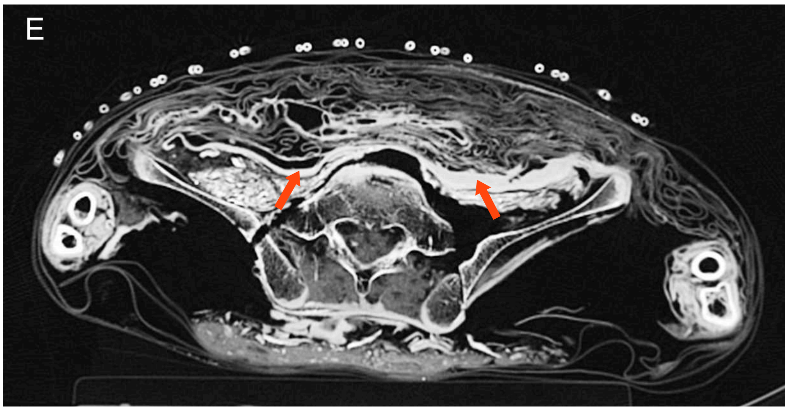

Supplement: S1 File — (PDF) [file pone.0316018.s005.pdf]
